# Advances in Technologies for Boron Removal from Water: A Comprehensive Review

**DOI:** 10.3390/ijerph191710671

**Published:** 2022-08-27

**Authors:** Xiaowei Liu, Congjin Xu, Peng Chen, Kexin Li, Qikun Zhou, Miaomaio Ye, Liang Zhang, Ye Lu

**Affiliations:** 1 Zhejiang Key Laboratory of Drinking Water Safety and Distribution Technology, Zhejiang University, Hangzhou 310058, China; 2 Ocean College, Zhejiang University, Hangzhou 310058, China; 3 Institute of Municipal Engineering, Zhejiang University, Hangzhou 310058, China; 4 Huzhou Water Group Co., Ltd., Huzhou 313000, China

**Keywords:** boron removal, water treatment integrated technology, prospective

## Abstract

Boron overabundance in aquatic environment raises severe concerns about the environment and human health because it is toxic to various crops and induces many human and animal diseases with long-term consequences. In response to the boron pollution of water resources and the difficulty of eliminating boron from water for production and living purposes, this article summarizes the progress in research on boron removal technology, addressing the following aspects: (1) the reasons for the difficulty of removing boron from water (boron chemistry); (2) ecological/biological toxicity and established regulations; (3) analysis of different existing processes (membrane processes, resin, adsorption, chemical precipitation, (electric) coagulation, extraction, and combined methods) in terms of their mechanisms, effectiveness, and limitations; (4) prospects for future studies and possible improvements in applicability and recyclability. The focus of this paper is thus to provide a comprehensive summary of reported deboronation processes to date, which will definitely identify directions for the development of boron removal technology in the future.

## 1. Introduction

**Figure 1:**
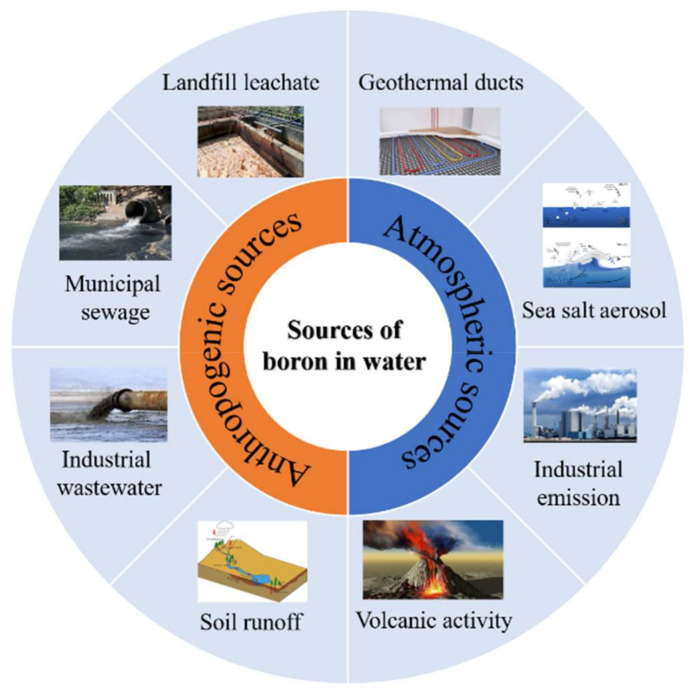
Various sources of boron contamination in water (reproduced from [10]).

Boron enters the aquatic environment through mineral extraction, coal burning, the discharge of wastewater with borax-produced detergents, boron fertilizer/pesticide applications, and the by-burning of boride-treated wood (Figure 1). The anthropogenic emissions of boron in surface waters from 2002 to 2016 grew from 0.44 to 0.65 TgB year^−1^. Due to the impact of human activities, increasing boron concentrations in ground waters and surface waters have been observed (Table 1). The existence of boron in aquatic environments can pose a serious threat to public health due to its toxicity [1]. Recently, water quality criteria have become more stringent because of the increased incidence of boron found in water resources due to the increasing global boron demand in industrial facilities. Therefore, the remediation of boron-spiked water has attracted a great deal of interest over the past few decades [2,3].

Deboronation methods reported include membrane separation, adsorption, (electro)coagulation and precipitation, extraction, and combinations of these methods. Many reviews have summarized the adsorption-based, coagulation-based, and membrane-based deboronation technologies and their applications to seawater or wastewater comprehensively [4,5,6,7,8,9]. However, these reviews paid little attention to methods based on novel synthetic or natural materials, chemical precipitation, and extraction. In particular, the integrated methods have not been thoroughly discussed.

**Table 1:** Content of boron in natural and contaminated waters (reproduced based on [11]).

Waters	Concentration (mg∙L^−1^)	Waters	Concentration (mg∙L^−1^)
Continental geothermal waters	1080	Rivers, Agricultural regions	0.193–0.387
Waters of active volcanic and geothermal activities	0.2–72	Rhine and Meuse rivers, The Netherlands	0.04–0.20
Rains, Germany, Switzerland	0.0003–0.007	Rivers, northern France	0.10 (<0.01–0.93)
Rains, Paris, France	0.002	Natural rivers, Liaoning, China	0.002–0.51
Rains, southern Asia	0.0003–0.009	Polluted rivers, Liaoning, China	0.039–25.1
Snowpack	0.002	Groundwater, average	0.04
Surface fresh	0.002	Mediterranean basin	3–13
River water, average	0.0003–0.002	Seawater, average	4.6

Therefore, this review aims to present a comparative analysis of different technologies that have been applied to boron removal from boron-spiked water, such as drinking water, wastewater, and seawater, with an assessment of their respective advantages and weaknesses. Additionally, a simple comparison is offered. Finally, from the findings of this overview of the current literature, the key challenges associated with current technologies and the prospects for future research are proposed.

## 2. Boron Overview

### 2.1. Reasons for the Difficulties of Boron Removal from Water

**Figure 2:**
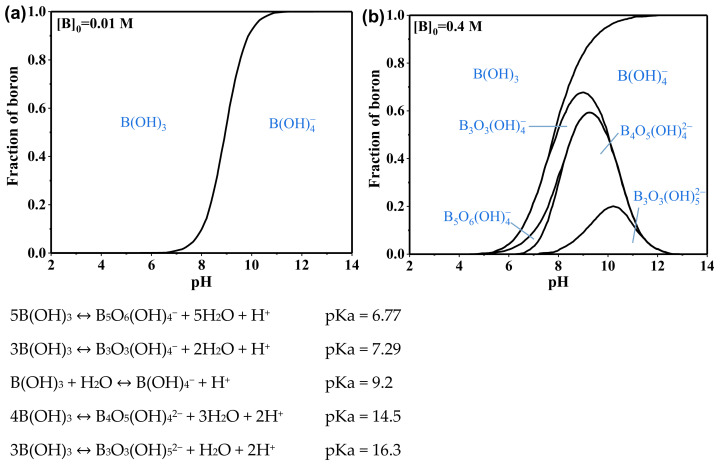
Distribution of boron species in aqueous solutions at different pH levels. (**a**) [B]_0_ = 0.01 M. (**b**) [B]_0_ = 0.4 M.

Boron is a trace element, which tends to form compounds by covalent bonds in nature due to its small atomic radius (about 0.85–0.90 Å) and high ionization energy (the first ionization potential is 8.296 eV). Boron in aqueous solutions usually exists in the form of polyborate anionic species, neutral boronic acid molecules, and singly negatively charged borates [8] (Figure 2). In the pH range of 6–9, boron at low concentrations mainly exists in the form of neutral boric acid molecules and single-charged borate anion in aqueous solution. The concentration of boron in seawater ranges from 0.5 to 6.0 mg·L^−1^, with an average concentration of about 4.6 mg·L^−1^ [12,13]. Boric acid is easily soluble in water (Table 2) and is not easily oxidized or decomposed. Due to the electron-deficient structure (Lewis acid) of boric acid and borate, they easily form complexes with polyhydroxy compounds in aqueous environments (Table 3). Uncharged boric acid has a molecular diameter of 2.75 angstroms and a molecular volume of 71.5 cubic angstroms, and its Stokes radius is only about 1.55 × 10^−10^ m [14], which is smaller than those of hydrated sodium ions (3.58 × 10^−10^ m) and hydrated chloride ions (3.58 × 10^−10^ m). One can also observe that the Stokes radius of boric acid is smaller than the aggregated pore size of most reverse osmosis (RO) membranes [15].

### 2.2. Toxic Effects of Boron

**Figure 3:**
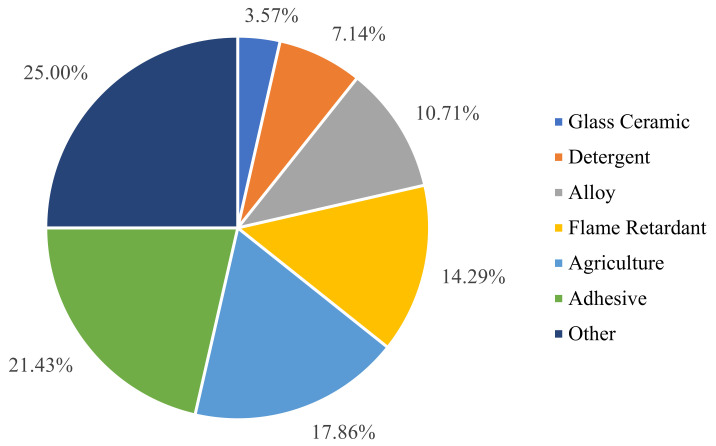
The proportion of boron consumption in various industries.

Boron is one of the seven essential trace elements in the natural world and is closely related to the survival and health of organisms. Boron intake, at a small amount, is beneficial to the growth and development of animals and humans, as well as the prevention of diseases [18]. It helps plant cell membrane to retain their structural and functional integrity and maintain a normal reproductive development and material transport [19,20]. Boron is not only an essential trace element for organisms, but also an important raw material for many industries, such as manufacturing and agriculture. According to the statistical data from 2020, borate alone has more than 300 applications, with more than three-quarters of global boron consumption being attributed to ceramics, detergents, fertilizers, and glass (Figure 3). In the production process, borate is the main component in the manufacturing of various special glasses and ceramics. The use of borosilicate in the production of advanced glass and glass fiber can improve the heat resistance and transparency of the glass, which is beneficial in improving its chemical stability and resistance to mechanical and thermal shock [21]. Incorporating boron into the glaze of porcelain can prevent deglazing and cracking and improve the product’s gloss and durability [22,23]. Adding borate to the porcelain body is beneficial in reducing energy consumption and improving the mechanical strength of the product. Boron is also used in the production of micro-alloyed forged steel and enhances the impact strength of the steel [24]. In the agricultural field, boron is used in products such as boron-containing fertilizers, herbicides, and pesticides [25]. In the nuclear industry, the isotope boron-10 can perform the function of chemical reaction compensation to control the rate of nuclear reactions in order to prevent explosions [26].

Boron is an essential micronutrient for the growth of many plants, but high levels of boron in irrigation water can lead to plant poisoning [27]. Excessive boron in drinking water can also cause harm to animals and humans. The dose toxicological effect of boron on animals is shown in Table 4. The European Union first identified the risks of boron to human health in 1993. Numerous studies have confirmed that excessive boron has adverse effects on the human organs, digestive system, immune system, blood components, and growth [28].

In response to the threat of boron to human health, the recommended value of boron intake (1–13 mg·d^−1^) for humans was given by the World Health Organization (WHO) [29], and the US Environmental Protection Agency set a limit of boron intake of ≤20 mg·d^−1^. A total of 40% of boron intake by humans comes from drinking water, and WHO further recommends a limit for boron (2.4 mg·L^−1^) in drinking water [5]. Different countries and regions around the world have formulated their own standards based on the recommendations of WHO according to their own contexts (Table 5).

## 3. Processes for Boron Removal from Water

### 3.1. Membrane Processes

The membrane process for water treatment employs a selective semipermeable membrane as a medium, and the solvent (or solvents) selectively passes through the membrane under different driving forces (e.g., pressure differences, temperature differences, and electric fields). The membrane processes for boron removal from water mainly include the reverse osmosis (RO) process, ion exchange membrane process (such as the electrodialysis (ED) process and Donnan dialysis (DD) process), forward osmosis (FO) process, and membrane distillation (MD) process.

#### 3.1.1. RO Process

**Figure 4:**
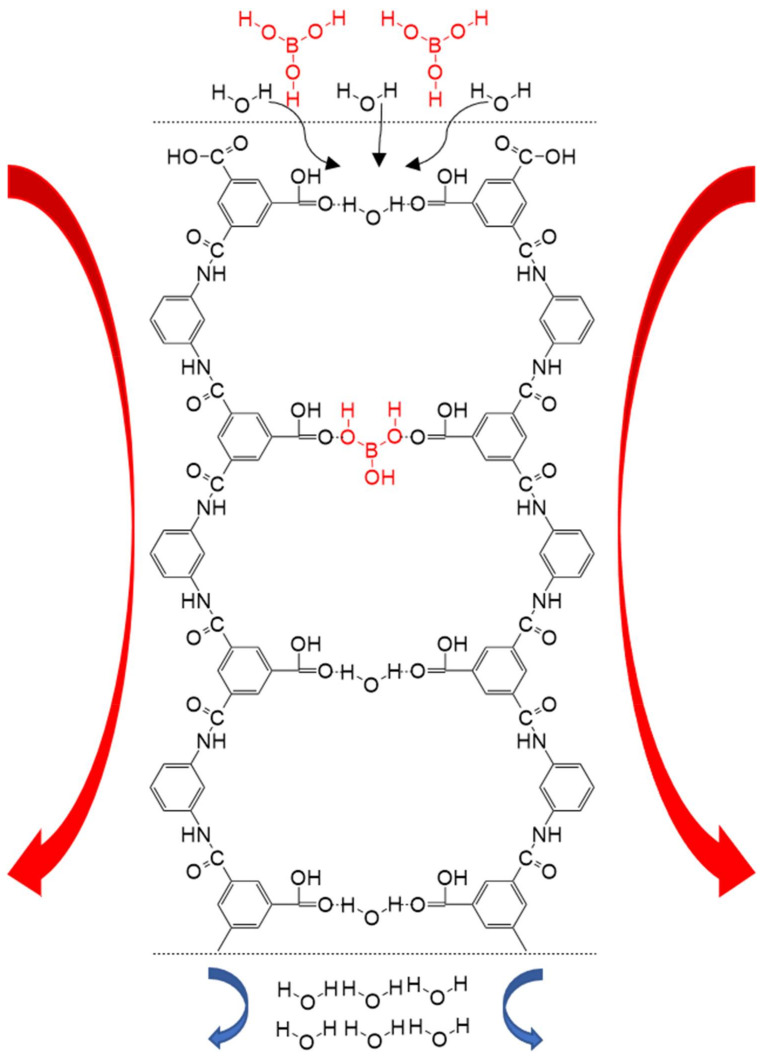
Transport of boron through the RO membrane.

The mode of transport of boric acid through the membrane is very similar to that of water. The hydroxyl group of boric acid easily forms hydrogen bonds with the bonding sites in the RO membrane (Figure 4), and then pressure drives the boric acid to permeate the RO membrane in the form of single molecule [32,33]. The Stokes radius of boric acid is only twice that of water, and boric acid has three hydroxyl groups, which can form six hydrogen bonds with water [34]. The hydrogen bond between the three hydroxyl groups of B(OH)_3_ and the water molecule in the membrane can enhance the binding and resistance of the water, augmenting the trade-off phenomenon compared to that of the water–salt transport [35].

The RO process can completely remove the boron under alkaline conditions, but the removal rate is only about 50% due to the dominance of non-ionized boric acid molecules under neutral or acidic conditions [36]. The efficiency of boron removal by RO is related to operating parameters, including the solution pH, initial boron concentration, salinity, temperature, recovery ratio, pressure, and feed flow rate, which are the most investigated factors. Despite these factors demonstrating important effects on the deboronation behavior in RO, increasing the pH and reducing the salinity and temperature are considered by consensus as effective strategies for augmenting the boron removal performance of RO.

Based on the physicochemical properties of boron and the fouling interception principle of the RO process, the boron removal performance of RO can be improved by adjusting the structure of the RO membrane. The regulation methods include modifying the physical structure of the membrane pores and altering the functional groups or charges on the membrane surface. Some scholars also divide the regulation methods into post-functionalization methods (directly introducing groups to the ready-made membranes), pre-functionalization methods (introducing groups during the membrane preparation process), combinations of the post-functionalization and pre-functionalization methods, and in situ polymerization methods [9]. Liu et al. (2019) [37] incorporated UiO-66 into the RO membrane to create a highly porous structure, which resulted in an 11% increase in boron removal. Li et al. (2020) [13] embedded 4-nitrobenzenesulfonyl chloride (NBS) into the RO membrane through the method of “swelling-embedding-shrinking.” The modification yielded an 11% increase in boron removal, which resulted from the steric hindrance increase and the synergistic repulsion of the –SO_3_H groups due to the implantation of NBS. Shultz et al. (2018) [38] proposed the use of hydrophobic long-chain aliphatic amine molecules (amylamine, n-octylamine, decylamine, and dodecylamine) to modify the RO membrane in situ. The permeated boron reduced by 2–4 times when the groups and charges of the RO membrane were altered by decylamine and dodecylamine. Polyisobutylene (PIB), m-phenylenediamine (MPD), and 1,3,5-benzenetricarbonyl trichloride (TMC) were also used as modifiers of the RO membrane through interfacial polymerization [39]. The modified RO membrane had a boron rejection rate of more than 90%. Hu et al. (2016) [40] achieved boron rejection enhancement by the interfacial polymerization of a novel sulfonated diamine monomer (4,4′-(1,2-ethanediyldiimino)bis (benzenesulfonic acid, EDBSA)) and trimesoyl chloride (TMC) on a poly(ether sulfone) (PES) substrate. Such a modification altered the membrane pore size, adjusted the surface hydrophilicity/hydrophobicity, and weakened the hydrogen bond strength.

Tu et al. (2013) [41] proved that adding polyols including glycerol, mannitol, or sorbitol to the feed solution can significantly improve the boron rejection of RO. The efficiency of specific polyol in improving the boron rejection was directly related to the stability constant of their complexation with the boron. Importantly, the presence of polyols does not cause any obvious membrane fouling. Dydo et al. (2014) [42] evaluated the effects of membrane type and different permeation fluxes, boron contents of the feeding water, alcohol/boron molar ratios, and pH values on the boron removal efficiency by RO in boron–polyol complex systems. Under relatively stable conditions (feed water boron content = 50 mg·L^−1^, pH = 10.0, permeation flux = 50 L·m^−^^2^·h^−^^1^, alcohol/boron molar ratio = 2), the boron rejection rate of the RO membrane SW-30 could reach about 99% in boron–D-mannitol complex systems.

#### 3.1.2. FO Process

**Figure 5:**
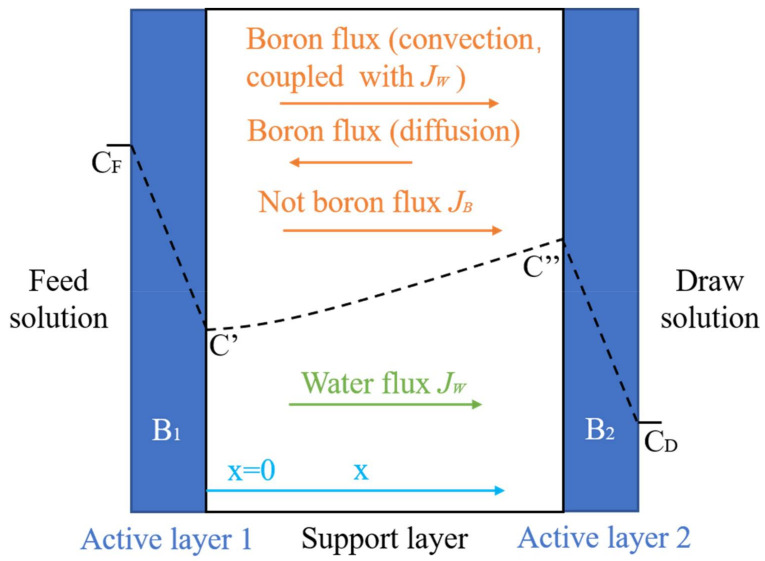
Transport of boron through the FO membrane.

The FO process is a new concentration-driven membrane separation technology, which relies on the osmotic pressure differences between each side of the selective osmosis membrane (Figure 5). The FO process is characterized by low energy consumption and high water recovery. The concentration polarization, membrane fouling, and the draw solution of reverse osmosis were barriers that had to be overcome for the application of the FO process. The boron removal efficiency of the FO process is affected by factors such as the membrane material, water flux, and pH value. To improve the boron rejection of the FO process, a great deal of effort has been put into developing novel membrane materials and draw solutions. Darwish et al. (2020) [43] studied the boron removal effect of three FO membranes (FTS H_2_O™ membrane, PSU membrane, and Aquaporin Inside™ membrane). The testing results indicated that the boron removal rate is closely related to the solution pH. When the pH value increased from 4 to 10, the boron removal rate increased from 12% to 90% for the FTS H_2_O™ membrane, from 8% to 84% for the PSU membrane, and from 8% to 76% for the Aquaporin Inside™ membrane. Wang et al. (2017) [44] and Fam et al. (2014) [45] also confirmed that the boron removal rate of the FO membrane depends on a high pH.

#### 3.1.3. ED Process and DD Process

**Figure 6:**
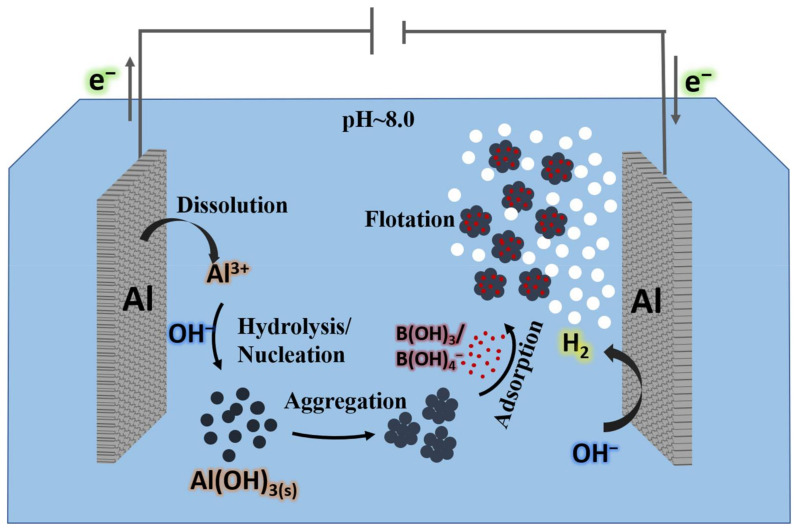
Diagram of the electrocoagulation of boron by Al electrodes.

In the ED process, the aqueous ions directionally migrate through the ion exchange membrane under the influence of a DC electric field to achieve separation (Figure 6). The process has the advantages of a flexible device design and application, convenient operation and maintenance, a long device service life, and a high recovery rate of raw water. The boron removal efficiency of the ED process is mainly affected by the membrane type, voltage, pH value, salinity, and flow rate. Sun et al. (2020) [46] prepared a novel anodic oxide membrane with quaternized graphene oxide-P84 composite. This anodic oxide membrane showed a better boron removal rate than the commercial membrane CJMA-3. Under the same conditions, the boron removal performance of the former was 25% higher than that of the latter. Dydo et al. (2013) [47] systematically investigated the effects of the initial boron concentration, membrane type, ion type, current density, and other parameters on the boron removal efficiency by the ED process. The initial boron concentration and current density were found to be the main influencing factors, followed by the ion type. The influence of the cation followed the order of K^+^ > Na^+^ > Ca^2+^ > Mg^2+^, and the anion was sorted as SO_4_^2−^ > NO^3−^ ≈ Cl^−^. On the basis of the ED process, the bipolar membrane electrodialysis (BPED) process was developed [48]. In BPED, the two sides of the ion-exchange membranes are bipolar (one side is a negative membrane and the other side is a positive membrane), and the middle is an anion exchange membrane with two chambers. In the first chamber, boric acid and hydroxide ions form tetrahydroxyborate ions. Under the influence of the current, the tetrahydroxyborate ions pass through the anion exchange membrane and enter the second chamber, forming a concentrated boron solution for the boron recovery. Compared with the ED process, the BPED process has more advantages in boron recycling. The ED process remains constrained in terms of its utility due to limitations such as membrane clogging, high cleaning frequency, and high operating costs.

The effectiveness of the borate transport across the anion exchange membranes in a DD process was demonstrated by Ayyldiz and Kara [49]. They also proved that the pH values of the feed and receiving solution, the type of membrane, the boron concentration in the feed solution, the presence of accompanying ions in the feed solution, and the type of carrier anion in the receiving solution all affected the boron flux in the DD process. Under the optimal conditions, the maximum boron flux could reach about 3500 μg·m^−2^·s^−^^1^. Trifi et al. (2021) [50] used the response surface methodology (RSM) to study the removal of boron from aqueous solution through the DD process. Under the optimal conditions ([B] = 66 mg·L^−1^, pH = 11.6 and [Cl^−^^1^] = 0.5 mg·L^−1^), 88.8% of boron could be removed by using an AFN membrane. Ping et al. (2015) [51] combined the DD process with microbial desalination cells (MDCs) to establish a new system for boron removal. When the initial boron concentration was 5 and 20 mg·L^−1^, the boron removal rate of this system was 60% and 52%, respectively.

#### 3.1.4. MD Process

The MD process is a thermally driven membrane separation technique, which has the advantages of a high removal efficiency of non-volatile substances. Because MD mainly relies on volatilization, the boron removal efficiency of the MD process is greatly affected by temperature and the salt concentration [27]. Compared to the RO process, boron removal by the MD process is less affected by pH [27,52]. Like ED, the MD process is still not useful because of its limitations of membrane fouling and high energy consumption, weakening its attractiveness. Hou et al. (2013) [53] applied a polyvinylidene fluoride (PVDF) flat-sheet membrane in the direct contact membrane distillation (DCMD) process to remove the boron, and they identified a high boron retention rate of about 99.8% across a wide range of feed boron concentrations. In another work, the DCMD process combined with commercial polypropylene (PP) membranes was used to treat highly saline, low-level radioactive wastewaters containing boron. A boron rejection of above 99.97% was maintained, even when the feed boron concentration reached 5000 mg·L^−1^ [54].

Among the above-mentioned membrane processes used for boron removal, the RO process is the most widely used (Table 6). However, there are problems with the RO process that still need to be solved, such as the membrane damage caused by high pH, membrane fouling, and concentrated water treatment. At present, some advances have been achieved in the development of high-efficiency boron removal membranes, but most of them are still far from practical application. Therefore, many scholars have proposed that membrane processes should be combined with other processes to achieve efficient boron removal.

### 3.2. Adsorption Technologies

The adsorption technology involves transferring the boron from the water to the surface of the adsorbent. According to the adsorption principle, adsorption can be divided into physisorption and chemisorption. Physisorption is mainly based on van der Waals forces, while chemisorption is mainly based on the formation of covalent or ionic bonds between the boron and functional groups on the surface of the adsorbent. In practical applications, chemisorption technology for boron removal is dominant due to its insensitivity to the concentrations of background anions and cations and its good selectivity [55]. Adsorption is often performed to treat high-concentration boron solutions, such as industrial wastewater and bittern. The concentration of boron in seawater is low and thus requires a long adsorption equilibrium time. Reported boron adsorbents include activated carbon, layered double hydroxides (LDHs), industrial waste materials (such as concrete particles [56]), natural materials (such as eggshells [57]), metal organic frameworks (MOFs), and porous aromatic frameworks (PAFs)), and other novel materials (such as zirconium–chitosan hydrogel beads [58]), etc.

#### 3.2.1. Carbon-Based Materials

Carbon-based materials, as adsorbents, have the advantages of high availability, being environmentally friendly, and high adsorption capability (Table 7). Common carbon-based materials include activated carbon (AC), graphene oxide (GO), and carbon nanotubes (CNT). Activated carbon possesses developed pores and a huge specific surface area and ranks among the most widely used adsorbents in the field of water treatment. Activated carbon is good at adsorbing non-polar substances and behaves poorly when adsorbing polar adsorbate, such as boron. Therefore, modification is needed in order for AC to enhance boron adsorption. Kluczka et al. (2019) [59] compared the boron adsorption performances of seven different activated carbons (WD Extra, WG-12, Filtrasorb 400, Norit SX2, Norit AZO, AquaSorb BP2, and CWZ-22) and found that Filtrasorb 400 (F400) had the best boron removal efficiency. To further increase its adsorption capability, F400 was modified with polyhydric chelates (mannitol, xylitol, and sodium gluconate). F400 modified with mannitol behaved the most effectively (1.5 mg·g^−1^). The improved boron removal efficiency resulted from the fact that the modification with polyhydric chelates increased the pore volume of the AC and increased the number of surface hydroxyl functional groups. Halim et al. (2013) [60] attempted to modify AC with curcumin. The maximum boron adsorption capacity of the modified AC was 5.0 mg·g^−1^, which was 4.4 mg·g^−1^ higher than that of the unmodified AC. Other attempts, such as modifications with calcium chloride, orthophosphoric acid, tartaric acid, citric acid, gluconic acid, and mannitol, were carried out [61]. In the static system, AC impregnated with tartaric acid had the strongest boron adsorption capacity, while in the dynamic system, AC impregnated with mannitol had the strongest boron adsorption capacity. Activated carbon is cheap, easy to obtain, and effective to use, but its modification brings extra costs, so that the economic benefit is not great.

Graphene oxide (GO) bears abundant hydrophilic groups (e.g., hydroxyl groups and epoxide carboxyl groups) on its surface, which gives it the potential to be used as a boron adsorbent. Chen et al. (2017) [62] applied nitrogen-doped graphene oxide (N-GO) to treat seawater with 5 mg·L^−1^ boron. The adsorption capacity was observed at 2.42 mg·g^−1^, corresponding to a boron rejection rate of 77.4%. According to the Langmuir adsorption isotherm, the maximum adsorption capacity of N-GO was 58.7 mg·g^−1^, placing it among the best boron absorbents reported thus far. This high adsorption capacity is mainly attributed to the large number of hydroxyl groups and nitrogen doping sites acting as adsorption sites. Hu et al. (2020) [63] prepared a boron adsorbent (GO/ZIF-67) with GO as the template. At 25 °C, the boron adsorption capacity of GO/ZIF-67 was determined to be 66.65 mg·g^−1^, which is much higher than that of ZIF-67 (26.31 mg·g^−1^). The boron adsorption sites of GO/ZIF-67 are identified as cobalt ions and hydroxyl groups.

Compared to conventional AC, the carbon nanotube (CNT) possesses a higher specific surface area, strength, flexibility, and structural homogeneity. Ismanto and Liu (2014) [64] compared the performances of boron adsorption by AC and CNT modified with polyvinyl alcohol (PVA). Due to the hydroxyl groups contained in the PVA molecular structure, the modification with PVA can increase the adsorption capacity of the adsorbent. However, the PVA modification caused an obvious blockage of the AC micropores, while the pores of the CNT were less blocked. The results also showed that the boron adsorption capacity of the PVA-modified AC and CNT increased with the increase in the pH value.

**Table 7:** Boron adsorption performances of different carbon-based materials adsorbents.

Adsorbent	Conditions	Equilibrium ^a^/Maximum ^b^ Adsorption Capacity	Refs.
F400	[B]_0_ = 30 mg·L^−1^, 24 h, pH = 7, adsorbent dose = 0.04 g·L^−1^, 20 ± 1 °C	^a^ 0.319 mg·g^−1^	[59]
WD Extra	[B]_0_ = 30 mg·L^−1^, 24 h, pH = 7, adsorbent dose = 0.04 g·L^−1^, 20 ± 1 °C	^a^ 0.152 mg·g^−1^	[59]
WG-12	[B]_0_ = 30 mg·L^−1^, 24 h, pH = 7, adsorbent dose = 0.04 g·L^−1^, 20 ± 1 °C	^a^ 0.144 mg·g^−1^	[59]
Norit SX2	[B]_0_ = 30 mg·L^−1^, 24 h, pH = 7, adsorbent dose = 0.04 g·L^−1^, 20 ± 1 °C	^a^ 0.238 mg·g^−1^	[59]
Norit AZO	[B]_0_ = 30 mg·L^−1^, 24 h, pH = 7, adsorbent dose = 0.04 g·L^−1^, 20 ± 1 °C	^a^ 0.191 mg·g^−1^	[59]
AquaSorb BP2	[B]_0_ = 30 mg·L^−1^, 24 h, pH = 7, adsorbent dose = 0.04 g·L^−1^, 20 ± 1 °C	^a^ 0.191 mg·g^−1^	[59]
CWZ-22	[B]_0_ = 30 mg·L^−1^, 24 h, pH = 7, adsorbent dose = 0.04 g·L^−1^, 20 ± 1 °C	^a^ 0.193 mg·g^−1^	[59]
F400 + mannitol	[B]_0_ = 60 mg·L^−1^, 4 h, pH = 7, adsorbent dose = 20 g·L^−1^, 25 °C	^a^ 1.50 mg·g^−1^	[59]
F400 + xylitol	[B]_0_ = 60 mg·L^−1^, 4 h, pH = 7, adsorbent dose = 20 g·L^−1^, 25 °C	^a^ 1.45 mg·g^−1^	[59]
F400 + sodium gluconate	[B]_0_ = 60 mg·L^−1^, 4 h, pH = 7, adsorbent dose = 20 g·L^−1^, 25 °C	^a^ 1.04 mg·g^−1^	[59]
Cur-AC	[B]_0_ = 1000 mg·L^−1^,2 h, pH = 5.5, adsorbent dose = 40 g·L^−1^, 25 °C	^b^ 5.0 mg·g^−1^	[60]
CWZ-30	[B]_0_ = 30 mg·L^−1^, 2 h, pH = 6, adsorbent dose = 20 g·L^−1^, 20 °C	^a^ 0.294 mg·g^−1^	[61]
CWZ-30 + glucose	[B]_0_ = 30 mg·L^−1^, 2 h, pH = 6, adsorbent dose = 20 g·L^−1^, 20 °C	^a^ 0.335 mg·g^−1^	[61]
CWZ-30 + CaCl_2_	[B]_0_ = 30 mg·L^−1^, 2 h, pH = 6, adsorbent dose = 20 g·L^−1^, 20 °C	^a^ 0.568 mg·g^−1^	[61]
CWZ-30 + citric acid	[B]_0_ = 30 mg·L^−1^, 2 h, pH = 6, adsorbent dose = 20 g·L^−1^, 20 °C	^a^ 0.671 mg·g^−1^	[61]
CWZ-30 + H_3_PO_4_	[B]_0_ = 30 mg·L^−1^, 2 h, pH = 6, adsorbent dose = 20 g·L^−1^, 20 °C	^a^ 0.384 mg·g^−1^	[61]
CWZ-30 + tartaric acid	[B]_0_ = 30 mg·L^−1^, 2 h, pH = 6, adsorbent dose = 20 g·L^−1^, 20 °C	^a^ 0.648 mg·g^−1^	[61]
CWZ-30 + salicylic acid	[B]_0_ = 30 mg·L^−1^, 2 h, pH = 6, adsorbent dose = 20 g·L^−1^, 20 °C	^a^ 0.325 mg·g^−1^	[61]
N-GO	[B]_0_ = 5 mg·L^−1^, 48 h, pH = 8.5, adsorbent dose = 1.6 g·L^−1^, 25 °C	^b^ 58.7 mg·g^−1^	[62]
GO/ZIF-67	pH = 11, 25 °C, adsorbent dose = 1 g·L^−1^	^b^ 66.65 mg·g^−1^	[63]
CNTs	[B]_0_ = 20 mg·L^−1^, 24 h, pH = 8.7, adsorbent dose = 4 g·L^−1^, 25 °C	^b^ 1.28 mg·g^−1^	[64]
PVA–CNTs	[B]_0_ = 20 mg·L^−1^, 24 h, pH = 8.7, adsorbent dose = 4 g·L^−1^, 25 °C	^b^ 1.19 mg·g^−1^	[64]

^a^: The equilibrium adsorption capacity is the adsorption capacity when the adsorption rate is equal to the desorption rate. ^b^: The maximum adsorption capacity is the ideal adsorption capacity that all adsorption sites are filled with adsorbate.

#### 3.2.2. Commercial Boron-Specific Resins and Fibers

The resin method achieves the selective removal of boron by coordination (for a neutral boric acid molecule) or ion exchange between the ortho-cis hydroxyl groups on the resin surface and the borate ions. Common commercial boron removal resins and fibers include Amberlite IRA743, Diaion CRB02, Purolite S108, etc., as shown in Table 8.

**Figure 7:**
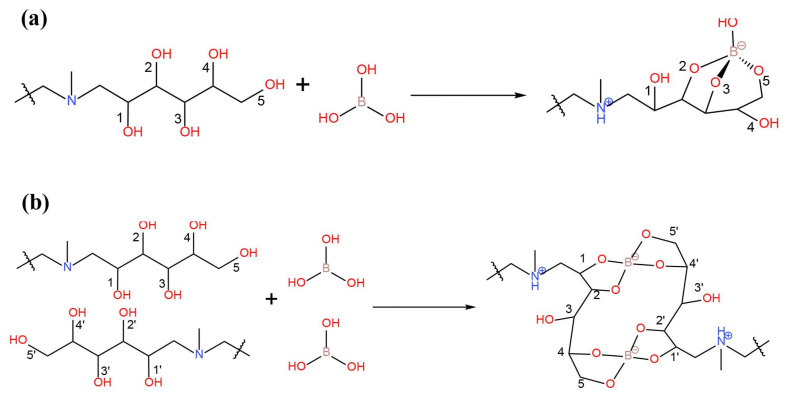
Representative scheme of boric acid chelation by N-methyl-D-glucosamine with (**a**) tridentate 2,3,5-isomer and (**b**) 1,2,4′,5′-4,5,1′,2′ bischelate. Reproduced based on [68].

The earliest report on the removal of boron by the resin method appeared in 1957 [30]. The researchers behind this work prepared a gel-type resin by reacting chloromethylated polystyrene with N-methyl-D-glucosamine (NMDG). NMDG has a structure of polyol and tertiary amine end groups, which has the ability to react with boron (Figure 7. NMDG resin is characterized by high selectivity, large adsorption capacity, and a fast adsorption speed, which have prompted its widespread use. At present, NMDG is still the most common functional monomer used for preparing boron removal resins (Table 8). In addition to using resin as the NMDG carrier, some manufacturers also use fiber as the carrier to replace the resins. Generally, these commercial resins and fibers show boron removal rates ranging from 93% to 98% [8].

Recepoğlu et al. (2018) [67] used an NMDG-modified Chelest fiber GRY-HW in a packed bed column to remove boron from geothermal bittern. It was discovered that the feed flow rate had a significant effect on the adsorption capacity of the Chelest fiber GRY-HW. When the feed flow rate decreased from 0.250 mL·min^−1^ to 0.125 mL·min^−1^, the adsorption capacity increased from 6.13 mg·g^−1^ to 12.07 mg·g^−1^. Ikeda et al. (2011) [69] successfully prepared chelate fibers by introducing an epoxy group-containing monomer, glycidyl methacrylate (GMA), to 6-nylon fibers by electron-beam-initiated graft polymerization, followed by N-methylglucamine (NMG) addition to the epoxy groups to form chelates. The results showed that, under the conditions of a boron solution concentration of 150 mg·L^−1^, the dynamic binding capacity of the chelating-fiber-packed bed was 2.5 times as high as that of the chelating-bead-packed (DIAION CRB05) bed. Ting et al. (2021) [70] successfully transplanted glycidyl methacrylate (GMA) onto 6-nylon fibers by the radiation-induced emulsion grafting method and posttreatment with NMDG. The obtained fiber adsorbent was tested using real industrial wastewater with high concentrations of ammonia and showed a boron adsorption capacity of 12.0 mg·g^−1^ at the feed space velocity of 20 h^−1^. These results showed that the prepared fiber adsorbent had good boron selectivity despite the existence of competing ionic pollutants in the wastewater. In addition, the adsorbent showed little adsorption capacity loss after five sorption/desorption regeneration cycles.

The boron removal efficiency of resin is related to factors such as particle size, dosage, contact time, the initial concentration of the boron, solution pH, and temperature [71]. As the resin particle size decreases, the adsorption contact area increases and the diffusion resistance of the boron decreases. Increasing the dosage of resin can also increase the total adsorption contact area. Higher solution temperatures can enhance the diffusion of the boric acid to the resin surface and the conversion of the boric acid to tetrahydroxyborate ions. In addition to the abovementioned commercial resins, a great deal of effort has been put into preparing resins based on commercial resins or developing new resins.

To improve the removal capability of boron-specific resin, a feasible strategy is to graft other functional groups onto the resin surface [72,73,74]. Based on atom transfer radical polymerization (ATRP), NMDG resin grafted with glycidyl methacrylate was prepared and obtained a saturated adsorption capacity of 20 mg·g^−1^ at the optimal grafting rate [75]. Compared with the NMDG resin without grafting, the boron adsorption capacity increased by 1.5 to 2 times. Wang et al. (2014) [76] developed a novel resin grafted with catechol functional groups, which showed a maximum adsorption capacity of 4.5 mg·g^−1^ at 25 °C. This resin did not exhibit a loss in boron removal ability after three cycles of regeneration applied with 10% AcOH after the adsorption saturation. Hussain et al. (2019) [77] studied an IX resin specially designed for boron removal. The IX resin had a strong boron removal ability (saturated adsorption capacity of 5 mg·g^−1^) at a neutral pH and was even very effective in the separation of boron at a low concentration. Its regeneration can be achieved by washing with 7% HCl and 4% NaOH solution. The characteristics of these modified boron-specific resins based on NMDG resins are summarized in Table 9.

The advantages of resin methods are simple operation, good selectivity, a high boron removal rate, insensitivity to salinity, and a high water yield. Its disadvantages are a limited exchange capacity, low mechanical strength, and the costs of regeneration.

#### 3.2.3. LDHs Adsorbents

**Figure 8:**
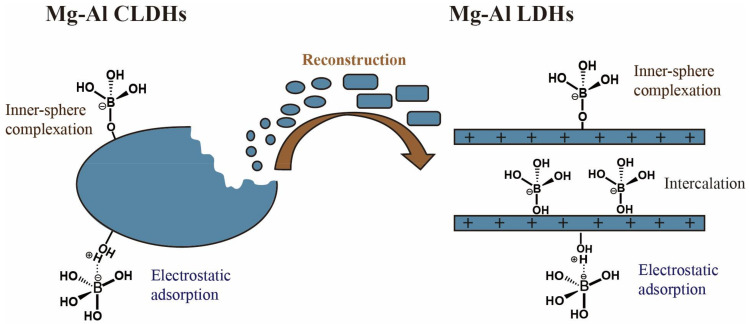
Mechanism of boron adsorption on Mg-Al layer-double hydroxides (LDHs). Reproduced based on [83].

LDHs are clay minerals with natural anion exchange characteristics. The boron adsorption mechanism of LDHs includes anion exchange and direct adsorption on the surface of the LDHs (Figure 8). An overview of the literature on the boron treatment of water using LDHs is presented in Table 10. Meng et al. (2018) [79] synthesized a novel CQDs/LDHs using 3D porous carbon quantum dots (CQDs) as the structure-directing agent. The CQDs/LDHs showed a saturated boron adsorption capacity of 19.5 mg·g^−1^. The boron removal mechanism includes anion exchange between the boron and nitrate and selective chemisorption between the boron and oxygen-rich functional groups of the CQDs/LDHs. Demirçivi et al. (2018) [80] developed a novel perlite-based boron adsorption method. This method used modifying agents (hexadecyl trimethyl ammonium bromide (HDTMA) and gallic acid (GA)) applied directly in the aqueous solution rather than pretreating the perlite with HDTMA and GA. Under the optimal conditions, the boron adsorption capacity of perlite-HDTMA and perlite-GA were 833.3 mg·g^−1^ and 2500 mg·g^−1^, respectively. The electrostatic effect caused the boron to be adsorbed on HDTMA and the esterification reaction resulted in the boron adsorption on GA. A hydrotalcite (HT) is an anionic clay mineral with the ability to remove boron from water. Shu et al. (2017) [81] proposed a strategy for accelerating the boron adsorption by hydrotalcite exfoliation and verified its effectiveness. Few-layered hydrotalcite (FHT) nanosheets were obtained by rinsing the coprecipitated HT with acetone. The speed of reaching the adsorption equilibrium using the FHT was about 10 times faster than that using the HT. These results indicated that the morphology of the 2D nanosheets not only contributed to the better dispersion of the FHT in the boron solution, but also provided more exposed active sites on its outer surface, which shortened the path of the boron transfer from the solution to the active adsorption sites and thus realized the rapid removal of the boron. It should be noted that the boron adsorption capacities of the FHT and HT are almost the same. Gao et al. (2017) [82] prepared Mg-Al-LDHs (I-LDH) by oxidative precipitation combined with ionothermal synthesis in a deep eutectic solvent (choline chloride: urea = 2:1) and compared its boron adsorption performance with that of LDH synthesized by the urea method (U-LDH). The prepared I-LDH showed a saturated adsorption capacity of 2.0 mM·g^−1^. Due to the narrow diameter distribution (10–40 nm) and monolayer structure (0.7 nm thickness), the interaction between the carbonate (competing anion) and metal layers was weaker. Therefore, I-LDH exhibited a higher boron adsorption capacity than U-LDH in the presence of co-existing anions. Furthermore, the calcined I-LDH (I-CLDH) also exhibited a better boron adsorption performance (Q_max_ = 7.2 mM·g^−1^). The main mechanism of the boron removal by I-LDH was ion exchange. Boron removal by I-CLDH consists of two stages. The first stage involves surface complexation and electrostatic attraction. The second stage involves immobilizing boric acid into Mg(OH)_2_ and using borate as an interlayer anionic species.

#### 3.2.4. Waste Industrial Materials

The preparation of boron adsorbents from waste industrial materials has attracted increasing attention in recent years. The boron adsorption capacities of waste industrial materials under the optimal operational conditions are summarized in Table 11. The main components of fly ash are silica and silicate, which exhibits a high alkalinity and thus favors neutral boric acid, dissociating into easily removed borate ions. Kluczka et al. (2014) [84] synthesized a new zeolite with fly ash as raw material, whose saturated adsorption capacity of boron reached 2.3 mg·g^−1^. The mechanism of boron removal by this synthesized zeolite was physisorption, and the adsorption kinetics conformed to a pseudo-second-order model. Babiker et al. (2019) [85] found that waste tire rubber (WTR) can also be used for boron adsorption. The equilibrium adsorption capacity of WTR was determined to be 16.7 ± 1.3 mg·g^−1^ at an initial boron concentration of 17.5 mg·L^−1^. The particle size of the WTR particles was negatively correlated with pH. The optimal particle size range was between 125–1000 μm, which corresponded to the pH value of 2 and showed the highest adsorption capacity. Iizuka et al. (2014) [56] used waste concrete particles to prepare a boron absorbent. The concrete particles could decrease 10 mg·L^−1^ boron to 2.2 mg·L^−1^. With high initial boron concentrations (100 and 300 mg·L^−1^ boron), the concrete-derived boron sorbent became incompetent in reducing the residual boron concentration below the recommended value. Ion exchange and the precipitation of calcium borate were considered to be the dominant aspects of the boron removal mechanism in the case of a low initial boron concentration and in the case of a high initial boron concentration. The heat treatment of the materials at 175 °C caused the initial boron removal rate to decrease but resulted in a lower residual level of boron after 1440 min compared to the untreated materials. Waste sepiolite obtained during the production of ornaments and tobacco pipes was confirmed as a feasible boron absorbent [86]. Under the conditions of pH = 10 and 20 °C, the non-activated waste sepiolite (NAWS) and hydrochloric acid-activated waste sepiolite (AWS) showed the highest boron removal performance, with a saturated adsorption capacity of 96.15 and 178.57 mg·g^−1^, respectively. The alkalinity and metal oxide composition of steelmaking slag also make it a suitable boron absorbent [87]. At initial boron concentrations lower than 6 mg·L^−1^, slag can reduce the boron to below the permissible levels for irrigation waters (<4 mg·L^−1^). The maximum boron adsorption capacity of steel slag was determined to be 145 mg·g^−1^.

#### 3.2.5. Natural Materials

**Figure 9:**
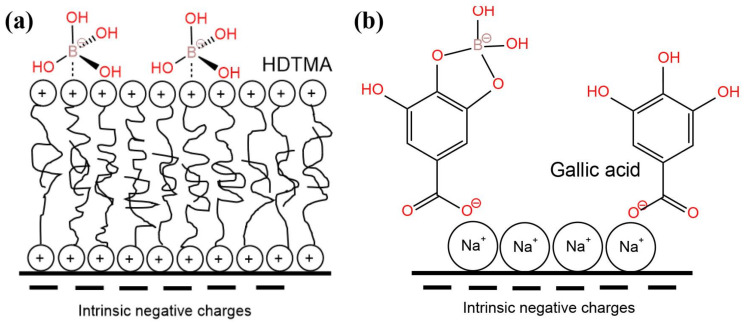
Scheme of the boron adsorption on vermiculate in the presence of (**a**) hexadecyltrimethylammonium bromide (HDTMA) and (**b**) gallic acid. Reproduced based on [80].

Compared with the abovementioned boron removal adsorbents, natural materials have obvious advantages in relation to their sources and prices. The boron adsorption capacities of natural materials under the optimal operational conditions are summarized in Table 12. Jalali et al. (2015) [88] used natural materials, such as bentonite, kaolinite, zeolite, waste calcite, wheat, rice, and walnut green shell, to prepare boron organic or mineral adsorbents and chemically modified them with ferric chloride. The adsorbents with the best performance were waste calcite and rice residue. Generally, organic adsorbents showed a higher boron adsorption capacity (5.59–9.26 mg·g^−1^) than mineral adsorbents (0.51–1.60 mg·g^−1^). Masindi et al. (2016) [89] used bentonite and magnesite powder to form composites with a good boron removal capacity (maximum adsorption capacity of 4 mg·g^−1^). The composites, at a dosage of 1 g, could decrease the boron concentration of mine leachates from 5 mg·L^−1^ to below 0.01 mg·L^−1^. The abundant boron adsorption sites of the composites can explain this phenomena. Demircivi and Saygili (2018, 2017) [80,90] modified vermiculite and perlite with hexadecyl trimethyl ammonium bromide (HDTMA) and gallic acid (GA). The maximum adsorption capacity of vermiculite-HDTMA and vermiculite-GA under the optimal conditions (pH = 11, 56.50 °C, initial boron concentration 7205 mg·L^−1^) was 258.13 and 152.4 mg·g^−1^, respectively. The adsorption capacity of perlite-HDTMA and perlite-GA under the optimal adsorption conditions was 833.3 and 2500 mg·g^−1^, respectively. HDTMA formed a double-layer structure with a negative charge on the surface, so that the borate anion was fixed by the electrostatic interaction, while GA was loaded on the clay surface through the electrostatic attraction at first, and then its diol functional group was complexed with the boron (Figure 9). Researchers have found that waste eggshells can also be used to remove boron. Al Ghouti et al. (2018) [57] prepared a boron adsorbent by calcining eggshell, whose saturated adsorption capacity was 31.06 mg·g^−1^ at 25 °C. This good boron adsorption capacity was attributed to the calcium oxide formed after the eggshell’s calcination, which not only adjusts the pH of water but also reacts with borate ions. The effective functional groups on the surface of the calcined eggshell were C=O and Ca-O. Al-Ghouti et al. (2018) [91] prepared a boron adsorbent with eggshell membrane (ESM) as a raw material, whose maximum adsorption capacity at the optimal pH (8.0) and temperature (25 °C) was 33.33 mg·g^−1^. The maximum adsorption capacity of eggshell membrane (MESM) esterified with methanol and hydrochloric acid at was still 33.33 mg·g^−1^, but the optimal pH was 4.0.

#### 3.2.6. Porous Organic Polymers (POPs)

Porous organic polymers (POPs) are a class of functional materials with the advantages of designability, easy functionalization, a high specific surface area, low density, uniform pore size, and excellent stability. At present, the reported POP adsorption materials for boron adsorption include metal-organic framework (MOF) materials, porous aromatic framework (PAF) materials, and three-dimensional ordered macroporous (3DOM) materials (Table 13).

MOF materials are open crystal frameworks with a permanent porosity, formed by the coordination of metal ions and polyatomic organic bridging ligands, which mainly rely on electrostatic interactions, π-π stacking, coordination bonds, and other effects to achieve boron adsorption. Lyu et al. (2017) [92] tested the boron removal performances of seven MOFs, ZIF-8, UiO-66, MIL-101(Cr), MIL-100(Cr), MIL-53(Cr), MIL-100(Fe) and MIL-96(Al). The results showed that all seven MOFs exhibited a good boron adsorption performance, and ZIF-8 had the best performance. ZIF-8 possessed an adsorption capacity as high as 247.44 mg·g^−1^ at 45 °C. Moreover, MOFs were also found to be competent in adsorbing boron at a high concentration. Zhang et al. (2019) [93] synthesized rhombic dodecahedral cobalt-based ZIF-67 at room temperature, which could reach a boron adsorption capacity as high as 579.80 mg·g^−1^. Five cycles of use caused the boron adsorption capacity of ZIF-67 to reduce by less than 6.0%. Lyu et al. (2017) [94] discovered that UiO-66 showed a boron adsorption capacity of 114.5 mg·g^−1^, and the adsorption equilibrium was reached in a short time (within 1 h). After four cycles of use, there was a minimal loss of boron adsorption capacity.

Porous aromatic frameworks (PAFs) are a relatively new class of porous network polymers, which have excellent characteristics, such as a high surface area, good stability in harsh environments, and an easily modified chemical structure. These characteristics give PAFs the potential to be used as a boron adsorbent. Kamcev et al. (2019) [72] used N-methyl-D-glucosamine (NMDG) as a modifier to obtain the adsorbents PAF-1-NMDG and P2-NMDG through a simple two-step synthesis. In the boric acid solution with an initial concentration of 19.4 mM·L^−1^, the saturated adsorption capacity of the two adsorbents was 1.70 and 1.56 mM·g^−1^, respectively, which was 70% and 56% higher than that of the commercial boron-selective resin Amberlite IRA743. In the simulated seawater with an initial boron concentration of 2.91 mg·L^−1^, the saturated adsorption capacity of these two adsorbents was 0.94 mM·g^−1^ and 0.90 mM·g^−1^, respectively, which was about 50% higher than that of Amberlite IRA743. The boron adsorption capacity of PAF-1-NMDG and P2-NMDG decreased due to the interference of other ions (such as chloride ion and nitrate ion) coexisting in the seawater. No obvious decrease in the adsorption performance was observed after 10 regeneration cycles.

Three-dimensional ordered macroporous (3DOM) materials have an interconnected macroporous structure, a high porosity (about 75%), and ultra-thin pore walls, with high-speed diffusion channels inside the material and a uniform surface for modification. Nan et al. (2018) [78] constructed three-dimensional ordered macroporous cross-linked poly(glycidyl methacrylate) (3DOM-CLPGMA) with water-soluble colloidal crystal templates (WS-CCTs) and further modified it with NMDG to prepare a super hydrophilic boron adsorbent for seawater treatment. This adsorbent, at 1 g·L^−1^, could reduce the boron from 4.24 mg·L^−1^ to 0.16 mg·L^−1^. After 10 adsorption regeneration cycles, there was a less than 15% loss in the adsorption capacity and little change in the ordered structure. By grafting NMDG onto the surface of chitosan beads (CTS), a boron adsorbent (CTS-NMDG) was prepared [95]. CTS-NMDG showed a high boron adsorption efficiency at a pH of 3–8, and the maximum adsorption capacity was 20.36 mg·g^−1^. The boron adsorption by CTS-NMDG was insensitive to general competing ions and thus made CTS-NMDG comparatively more selective compared to CTS.

#### 3.2.7. Metal Oxide-Based Adsorbents

**Figure 10:**
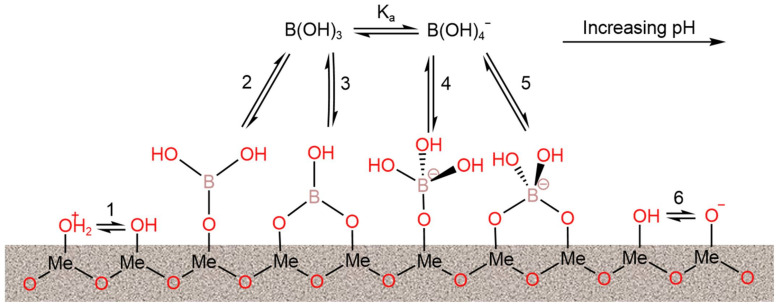
Representative mechanism of boron adsorption on the hydrous surface of metal oxides. Surface group deprotonation: reactions 1 and 6; monodentate complexation: reactions 2 and 4; bidentate complexation: reactions 3 and 5. Reproduced based on [80].

Metal oxide removes pollutants, such as phosphates, dyes, and organic compounds, by adsorption/coprecipitation, redox reaction, and electrostatic interaction. The mechanism of boron removal by metal oxide is considered to be one of adsorption (Figure 10). The boron adsorption capacities of reported metal oxide-based adsorbents under the optimal operational conditions are summarized in Table 14. MgO can absorb boron from H_3_BO_3_ solution through the electrostatic attraction between the positively charged MgO and B(OH)_4_^−^ [96]. The maximum adsorption capacity of MgO can reach 21.5 mM·g^−1^. Saturated MgO can be regenerated using an NaOH solution, but, in the experiments, the adsorption capacity of the regenerated MgO was found to decrease after regeneration. When the structure of MgO was regulated from three-dimensional particles to two-dimensional nanosheets, an improved adsorption performance was obtained [97]. MgO nanosheets prepared by the ultrasonic method, with a specific surface area ranging from 79 m^2^·g^−1^ to 168 m^2^·g^−1^, showed a maximum boron adsorption capacity ranging from 39.7 mg·g^−1^ to 86.7 mg·g^−1^. In order to further improve the boron adsorption capacity of metal oxide-based adsorbents, composite metal oxides were proposed for application as adsorbents in boron adsorption. Demey et al. (2019) [98] prepared a boron adsorbent (CAAI) based on calcium alginate and Al_2_O_3_. CAAI exhibited an adsorption capacity of 5.21 mM·g^−1^, which was much greater than that of the original alumina oxides (0.59 mM·g^−1^). Kluczka et al. (2017) [99] combined novel bio-composite chitosan beads (CTS) with metal oxide nanoparticles (TiO_2_, Cr_2_O_3_, and Fe_3_O_4_) to separate boron from aqueous solution. The adsorption equilibrium of the three composite absorbents (TiO_2_-CTS, and Cr_2_O_3_-CTS) was reached within 5 min, and their maximum adsorption capacity ranged from 3.52 to 4.42 mg·g^−1^.

#### 3.2.8. Other Materials

In addition to the abovementioned boron adsorption materials, a great deal of effort has also been made in searching for novel boron adsorption materials (Table 15). These boron adsorbents feature properties such as magnetic separation, a super specific surface area, environmentally friendly status, and little or no secondary pollution. Silica coated magnetic nanoparticles functionalized with NMDG were synthesized through direct coupling (M-NMDG) and click chemistry (M-TACA) methods [100]. The saturated adsorption capacity of M-NMDG and M-TACA was 6.68 mg·g^−1^ and 13.44 mg·g^−1^, respectively. To obtain rapid boron removal from water, a mesoporous nanosponge was prepared and modified with cis-diols [101]. Different cross-linking agents were used to adjust the surface morphology and porous structure of the cyclodextrin (CD) scaffold nanosponge, and then, the Staudinger reaction was carried out to generate amino groups on the primary surface of the CD for subsequent reaction with D-(+)-gluconic acid δ-lactone, which successfully immobilized the high-density cis-diols on the surface. The boron adsorption capacity of the mesoporous nanosponge was comparable to that of the commercial resin Amberlite IRA74. Luo et al. (2020) [55] synthesized two sponge-like multifunctional polymers based on a cyclodextrin skeleton with a saturated adsorption capacity of 31.1 mg·g^−1^ and 20.5 mg·g^−1^, respectively. Another work of Luo et al. (2020) [102] synthesized two new boron adsorbents, poly(glycidyl methacrylate-trimethylolpropane trimethacrylate)-ethylenediamine-glycidol (P(GMA-co-TRIM)-EN-PG) and poly(glycidyl methacrylate-trimethylolpropane trimethacrylate)-triethylenediamine-glycidyl (P(GMA-co-TRIM)-TETA-PG). The boron adsorption capacity of P(GMA-co-TRIM)-EN-PG and P(GMA-co-TRIM)-TETA-PG was determined to be 29.2 mg·g^−1^ and 23.3 mg·g^−1^, respectively. Another attempt was made to synthesize zirconium-chitosan (Zr-CTS) hydrogel beads for boron removal [58]. The Zr-CTS hydrogel beads exhibited a saturated boron adsorption capacity of 24.5 mg·g^−1^ at a pH of 6–7 and had a strong regeneration ability and convenient molding. The boron adsorption on the Zr-CTS hydrogel beads was achieved through the complexation of boron with the hydroxyl groups of zirconium oxides. To ensure simple separation, organic boron absorbent (Gum arabic) was immobilized on hollow silica spheres [103]. These functionalized hollow silica spheres obtained a maximum boron adsorption capacity of 44.3 mg·g^−1^. Sun et al. (2018) [74] tried to immobilize NMDG on biomass carbonaceous aerogels and obtained a novel boron adsorbent, CA@KH-550@EPH@NMDG (CKEN). The boron adsorption capacity of CKEN reached 31.8 mg·g^−1^. CKEN had a three-dimensional cross-staggered structure, and its surface contained a large number of hydroxyl groups, which facilitate boron adsorption. Its excellent reusability further increased the credentials of CKEN as boron absorbent. These new adsorbents have great application potential, but they are currently in the stage of laboratory research, and there have been no cases of commercial application [104].

### 3.3. Chemical Precipitation and (Electric) Coagulation

The conventional chemical precipitation method is suitable for treating high-concentration boron-containing water. By adding precipitants, boron is converted into insoluble borate to achieve its separation from water [105]. Inorganic boron precipitants mainly include calcium oxide, zirconium compounds, metal salts, and their mixtures. Commonly used organic boron precipitants include polyvinyl alcohol, hydroxycarboxylic acid, polyethylene glycol, etc. [106]. Tang et al. (1994) [107] used lime milk to precipitate boron in brine and found that 31.5 g·L^−1^ boron could be removed, equating to 71.4%. When polyvinyl alcohol was used to treat boron-containing water (150 mg·L^−1^), 60.0% of the boron was eliminated [108]. The disadvantages of the precipitation method are the high consumption of the precipitants and the production of chemical sludge. The chemical sludge requires further treatment, which brings with it extra costs. In addition, the pH of the treated water is usually high, making the water unsuitable for reuse.

Shih et al. (2014) [109] proposed a chemical oxidation precipitation (COP) method for boron removal. This method used peroxide to convert boron into perborate, which was easily precipitated by the precipitants (metal salts). In the case of COP technology, barium salt is the most effective metal salt precipitant. Under milder conditions (room temperature, pH 10), COP, using hydrogen peroxide (H_2_O_2_) to pretreat the boric acid, and precipitating the perborate with barium ions (Ba^2+^), effected a 98.5% boron removal at [H_2_O_2_]/[B] = 2 and [Ba]/[B] = 1 [110].

Conventional coagulation (CC) is one of the most frequently applied physicochemical processes for water and wastewater treatment. This class of methods add coagulants, such as aluminum or ferrum salts, to the water and produce flocs to trap boron, followed by solid–liquid separation through co-precipitation. The CC method is preferred for treating boron-containing waters with multiple pollutants. Its limitations, such as high operational costs resulting from a high chemical consumption and the production of large amounts of sludge, weaken its attraction. In the context of its application in deboronation, several studies have proven that this technique does not provide a satisfactory removal efficiency. Coagulation using aluminum chloride as the coagulant only had a boron removal rate of 24% at a pH of 8 [111]. Another work also found that the coagulation of water with 5 mg·L^−1^ boron showed a low boron removal efficiency (<10%) with an aluminum dosage of 30 mg·L^−1^ [112].

Boron removal by electrocoagulation (EC) is effected through the in situ generation of coagulants (corrosion of anode) [113]. During EC, parameters such as pH, current density (CD), interelectrode distance, conductivity, initial boron concentration, and anions co-existing with the boron are essential and influence the treatment efficiency. Generally, EC performs better than conventional coagulation in boron removal. An EC system with aluminum as an anode was applied to treat water with 100 mg·L^−1^ boron and had a 70% removal rate under the optimal conditions (pH value of 8, electrode distance of 10 mm, reaction time of 60 min, and current density of 5.5 mA·cm^−2^) [114]. Chen et al. (2020) [115] also confirmed the feasibility of boron removal by the EC system with aluminum as the anode (EC-Al). It was found that amorphous aluminum formed at a low current intensity showed a better boron adsorption capacity than amorphous aluminum formed at a high current intensity. However, problems related to EC, such as the consumption of the electrode plates and generation of sedimentary sludge, should be considered. The EC is not suitable for application to boron-rich streams, as it removes boron via the adsorption by the produced metal hydroxide. Güven et al. (2018) [116] studied the efficiency of EC-Al for removing high-concentration boron in surface water. For the waters with three initial boron concentrations of 1998 mg·L^−1^, 998 mg·L^−1^, and 500 mg·L^−1^, the boron removal rates were 33%, 46.5%, and 55%, respectively, after a 90 min reaction time (pH = 8.5, 10 mA·cm^−2^).

Changing the anode is a valid method for enhancing boron removal by EC. For example, EC-Ni can remove 99.2% of boron from a 10 mg·L^−1^ boron solution within 2 h at pH = 8 and a current density of 1.25 mA·cm^−2^ [117], while EC-Fe/Ni can remove 95% of boron from a 10 mg·L^−1^ boron solution within a shorter reaction time (60 min) at a pH of 8 and current density of 3.75 mA·cm^−2^ [118]. The type and concentration of the electrolyte are also important factors affecting the boron removal efficiency by EC. Widhiastuti et al. (2018) [118] demonstrated that various spinel ferrites (MFe_2_O_4_) can be generated using transition metal (nickel, cobalt, and cuprum) salts as electrolytes in EC-Fe. The boron removal efficiency was positively correlated with the maximum adsorption capacity of the spinel ferrites. NaCl was considered as an ideal electrolyte for EC-Al because it can promote anodic dissolution through pitting and complexation [119]. Compared to the test carried out without adding an electrolyte (corresponding to a boron removal rate of 46.5%), the addition of NaCl, Na_2_SO_4_, and KCl increased the removal rate up to 50.6%, 49.3%, and 50.4%, respectively, under the same conditions [116]. The electrolyte concentration showed a positive relationship with the boron removal efficiency, and the change in the solution resistance was the reason for this [120]. The current density (CD) is another important factor related to the efficiency of boron removal by EC. The boron removal rate increased from 43% to 74% when raising the CD from 2.5 to 5.0 mA·cm^−2^, and further raising CD to 6.25 mA·cm^−2^ did not improve the boron removal efficiency significantly. In addition, the cost of the operation increased by 2.7 times when the CD was raised from 2.5 to 6.25 mA·cm^−2^ in EC-Al [121]. In another two studies, the boron removal efficiency increased with the increase in the CD, ranging from 0.18 to 6 mA·cm^−2^ [122,123]. The interelectrode distance also plays an important role in boron removal by EC. The boron removal efficiency increased from 73% to 92% when the interelectrode distance was decreased from 2 to 0.5 cm after 150 min of electrolysis in EC-Al [124]. A similar phenomenon was observed by [125], who found that the boron removal decreased from 84% to 72.7% using an aluminum electrode and 76% to 61.3% using an iron electrode when increasing the electrode distance from 0.5 to 1.5 cm. Therefore, decreasing the interelectrode distance is a valid method for enhancing the boron removal efficiency by EC. Boron removal by EC is greatly influenced by pH. Massara et al. (2018) [126] studied the boron removal efficiency at different initial pH levels (4, 5, 6, 7.45, and 9). The results showed that the highest boron removal (67%) was obtained at a pH of 6. The boron removal efficiency by EC-Al with different pH levels, ranging from 2 to 8, in the presence of coexisting ion arsenic was studied in another work [127]. The highest removal efficiencies were observed at an initial pH of 4.0 for the boron. Differences in the reactor design of EC, such as the monopolar (MEC) or bipolar (BEC) modes, and the batch system or continuous system, are also unignored factors related to boron removal. Several tests run in the MEC and BEC modes were carried out under the same conditions, and the highest boron removal efficiency (96%) was achieved using the BEC mode within 150 min of the treatment time [124]. An overview of the literature on the boron treatment of water using the chemical precipitation method and (electric) coagulation is presented in Table 16.

### 3.4. Extraction Method

The principle of the extraction method is to use an organic solvent, containing o-dihydroxyl groups and being immiscible with water, as an extractant to react with boron and to transfer the boron from the water phase to the organic solvent phase. The greater the difference between the partition coefficients of the complexes in the two solvents was, the better the separation efficiency was. The key aim of this method is to identify an extractant with a low toxicity and high selectivity. Reported extractants include 2-ethyl-1,3-hexanediol (EHD), 2-chloro-4-(1,1,3,3-tetramethylbutyl)-6-methylphenol (CTMP), 2,2,4-trimethyl-1,3-pentanediol (TMPD), 2-butyl-2-ethyl-l,3-propanediol (BEPD), N,N-bis(2,3-dihydroxypropyl) octadecylamine (BPO), 2-butyl-1-n-octanol, 2-ethylhexanol, and 1,3 diolic compounds [130,131,132,133,134,135,136,137,138]. Ayers et al. (1981) [130] studied the extraction efficiency of boron with 2-ethyl-1,3-hexanediol (EHD) and 2-chloro-4-(1,1,3,3-tetramethylbutyl)-6-methylphenol (CTMP). The extraction efficiency of the EHD/CTMP mixed reagent (concentration ratio of 1:1) was significantly enhanced in the pH range of 8–12, while the extraction efficiency of the mixed reagent with the EHD/CTMP concentration ratio of 3:1 was basically independent of the pH. The extraction of boron is largely unaffected by the type of cation (Na^+^, Ca^2+^ or Mg^2+^) used. Biçak et al. (2007) [139] demonstrated that a solution of 2-ethylhexanol containing N,N-bis(2,3-dihydroxypropyl) octadecylamine (BPO) was very effective in extracting boronic acid from aqueous solutions. The two vicinal-diol functions of BPO make it a versatile reagent for boron extraction, and its long aliphatic chains provide solubility when used in organic solvents. Using 0.1 M BPO organic solution to extract 0.1 M boric acid solution with the same volume, the extraction rate has been shown to reach 63.1%. Complexed boron can be recovered from the organic phase by treatment with 2 M H_2_SO_4_ solution. The extraction method is more suitable for the recovery and utilization of boron, as it will cause the loss of the extractant and the secondary pollution of the water if it is simply used to remove the boron. An overview of the literature on the boron treatment of water using the extraction method is presented in Table 17.

### 3.5. Capacitive Deionization (CDI) Process and Electrodeionization (EDI) Process

The feasibility of boron removal by the capacitive deionization process has been confirmed. The working principle of this method can be divided into two stages. The first stage involves the dissociation of the boric acid on the negative electrode, and the second stage involves the electro-adsorption of borate ions on the positive electrode. The boron removal rate of the CDI process is only about 30% [140]. Thus, an integrated UF membrane and membrane CDI (MCDI) system was developed, which showed an excellent boron removal performance in the range of 96–100% [141]. Continuous electrodeionization (CEDI) is very effective in the treatment of low-conductivity wastewater; therefore, it was investigated in its capacity to remove high-concentration boric acid. The experiments indicated that CEDI showed a low boron removal efficiency (about 26%) [142]. The boron-selective resin-filled electrodeionization (BSR-EDI) process was then proposed and used [143]. The applied potential and feed flow rate had significant effects on the boron removal in BSR-EDI, which obtained a boron removal rate as high as 94%. Arar et al. (2013) [144] investigated the hybrid process combining RO and EDI in its capacity to remove boron from geothermal water. The results showed that a layered bed configuration of the EDI system at a 40 V of voltage was able to reduce the boron from 5.9 mg·L^−1^ to 0.4 mg·L^−1^.

### 3.6. Integrated Methods

In view of the limitations of the sole application of membrane separation, adsorption, and coagulation, the combination of multiple technologies has been proposed for overcoming the shortcomings of single technologies and further improving the boron removal efficiency. The combination of the resin method and membrane processes is the most widely studied among the integrated methods. Boron-specific resin can be combined with a microfiltration membrane to improve the boron removal efficiency, in a method known as the adsorption-microfiltration (AMF) process. The boron removal by the AMF process can be divided into the following four main steps: (1) the resin absorbs the boron in the solution; (2) the microfiltration membrane separates the saturated resin; (3) the saturated resin on the microfiltration membrane is eluted; (4) resin recovery and recycling. Darwish et al. (2017) [145] studied the combination of Amberlite IRA743 resin and the microfiltration membrane and found that the boron removal rate was 96–99%. The boron-saturated resin particles could be effectively separated by the microfiltration membrane, but the resin itself could cause membrane fouling. Therefore, the mitigation of resin-induced membrane fouling and resin regeneration have become the focus of follow-up research. In addition, the size of the existing resins also limits the use of AMF; thus, reducing the particle size of the resins to increase the adsorption contact area is another focus of research. In response to the abovementioned problems of AMF, the researchers proposed a method for achieving resin regeneration and membrane regeneration in situ, using acid (HCl) to remove the boron from the resin, then neutralizing the resin with an alkaline solution (NaOH) to achieve the resin regeneration, and finally washing the membrane with acid (1.85% HCl), water, alkali (5% NaOH), and water, in turn, to achieve the membrane regeneration [146].

In addition, polymer-enhanced ultrafiltration (PEUF) boron removal technology has also received great attention in recent years. This technology converts boron into large-sized complexes by adding water-soluble high polymers to boron-containing water, which is easily retained by an ultrafiltration membrane. The reported water-soluble high polymers include polyvinyl alcohol, polyethylene, a new hydroxyl-terminated poly(ethyleneimine) (HPEI) polymer [147], and poly(vinyl amino-N,N′-bis-propane diol)(GPVA) [122], etc. The key function of PEUF boron removal technology lies in the control of the membrane fouling and the regeneration of polymers. Some scholars have used micelle-enhanced ultrafiltration (MEUF) as the RO pretreatment unit and obtained a 99% boron removal efficiency [148]. The working principle of this method is that surfactants are added to the solution, and when the surfactant concentration exceeds the critical micelle concentration, the monomer aggregates to form large transparent micelles, which form bonds with the boron, and the micelles are retained by ultrafiltration so as to achieve the boron removal. The combined process of MEUF with RO removes most of the boron in the ultrafiltration stage and focuses on desalination in the RO stage, so that the boron removal and desalination can be realized at the same time. Alharati et al. (2018) [149] carried out boron removal from seawater using a mixed ion exchange resin/microfiltration process without the continuous addition of resin. Under the experimental conditions of an Amberlite IRA743 resin dosage of 3.33 g·L^−1^ and microfiltration membrane with a pore size of 0.1μm, the boron in seawater was almost completely removed.

The combination of different membrane processes also offers a strategy for enhancing boron removal. Liu (2010) [150] achieved a final effluent boron concentration reduced to 0.84 mg·L^−1^ under the conditions of double-stage nanofiltration, a second-stage influent pH of 10.5, and an influent boron concentration of 3.87 mg·L^−1^. Under the conditions of two-stage nanofiltration with sorbitol complex reaction, a second-stage influent pH of 9.8, and an influent boron concentration of 2.174 mg·L^−1^, the final effluent boron concentration was controlled below 0.1 mg·L^−1^. Landsman et al. (2020) [151] found that ED, as a pretreatment process for NF and RO, could increase the boron rejection rates of NF and RO units by 10% and 20%, respectively. It was speculated that the ED treatment increased the electrostatic repulsion of the borate. The two-stage RO (SWRO-SWRO) process is widely used for seawater desalination, where the alkalinity of the first-stage RO filtrate is increased, resulting in the boron removal rate of the solution passing through the second-stage RO membrane being greater than 90% [77]. Ban et al. (2019) [152] further employed the combined boron removal process using an FO membrane and RO membrane (SWFO-SWRO) and found that the boron concentration could be reduced from 5 mg·L^−1^ to 0.4 mg·L^−1^ without any pH adjustment. Kayaci et al. (2020) [153] proposed a hybrid process combining multi-stage RO in sequence, low pressure membranes (LPMS), and a countercurrent membrane circulation (CMCR), which recycled the LPMS concentrate stream as part of the RO feed to improve the overall recovery. The osmotic pressure difference in the CMCR was reduced by the backflow of the osmotic fluid and the retentate liquid in the CMCR, thereby reducing the concentration differences between the stages and improving the energy efficiency. Compared with the RO process, this process was found to reduce the boron content of seawater from 10 mg·L^−1^ to 0.5 mg·L^−1^ without any pH adjustment.

In order to achieve the desalination and boron removal, many seawater desalination projects have developed the brackish water reverse osmosis (BWRO) process on the basis of SWRO, forming a typical SWRO + BWRO desalination and boron removal process. The boron removal efficiency rate of the SWRO + BWRO process, in a real operational context, can reach over 91%, and the final boron concentration in the produced water can meet the requirements of the water quality standards [154]. Another method for improving the boron removal efficiency of SWRO is to add boron-selective resin (BSR) on the basis of single-stage SWRO. Generally, due to the high selectivity of BSR, BSR can almost remove all the boron in water; thus, the removal rate of the boric acid can reach over 99%. The SWRO + BWRO + BSR desalination and boron removal process involves combining the two abovementioned processes. In this process, BWRO does not need to operate at a high pH value, which can save high costs incurred by purchasing chemicals, and the boron removal rate can reach over 92%. The SWRO + BWRO + BSR process is suitable for areas where desalinated water is the main source of drinking water.

## 4. Conclusions

This paper comprehensively reviewed the recent progress in the boron-removing technologies in terms of their principles, efficiency, characteristics, and applicable conditions. Based on the collected information, the following conclusions and recommendations can be drawn regarding the design and the optimization of technologies shouldering the task of boron removal:

(1) The RO process is a suitable technology for seawater desalination along with boron restriction. Nevertheless, the need for multiple RO stages to decrease the boron concentration below recommended standard poses a major restriction and increases the investment costs. Consequently, the combination of the RO process with other processes, such as adsorption or a membrane, such as ED, EDI, or MD, or even the EC process, is recommended.
(2) Adsorption techniques are only efficient for solutions with low boron concentrations and mineral concentrations when the goal is to prevent repeated regeneration operations. To overcome this limitation and improve the applicability of these techniques, developing novel magnetic porous support materials, in which the boron-specific chelating functional groups are embedded, is encouraged. Furthermore, with respect to the novel adsorption materials, assessments of their risks to human health should also receive more attention.
(3) Coagulation, electrocoagulation, and direct chemical precipitation, in essence, involve transforming the dissolved boron into undissolved boron-bearing solids, which immobilizes the boron inside their chemical structure. This class of methods are characterized by the excessive dosage of the chemicals; thus, it is suggested that, when the target is to remove multiple pollutants, including boron, researchers should weigh up the input costs and output benefits.
(4) As regards the integration methods, RO separation combined with coagulation, MF/UF combined with adsorption, and complexing membrane filtration (CMF) are considered to be economically, ecologically profitable, and promising techniques.
(5) Regarding the recycle of the treated boron, the boron immobilized by the adsorbents with a poor capacity for regeneration, or in the flocs from EC and co-precipitation, are expected to be difficult to recycle. More effort should be put into developing technologies with a high boron selectivity and high boron recyclability.

## Figures and Tables

**Table 2:** Solubility of boric acid in water at various temperatures (Adapted with permission from Ref. [16]. 2013, Elsevier).

Temperature (°C)	Solubility (Molar)	Temperature (°C)	Solubility (Molar)
0	0.4304	60	2.3961
10	0.5776	70	2.7067
20	0.8154	80	3.8424
30	1.0678	90	4.9151
40	1.4108	100	6.5119
50	1.8670		

**Table 3:** Equilibrium constants for boric complexes with polyols (Adapted with permission from Ref. [17]. 1997, Springer).

Polyol	k_1_ (L·mol^−1^)	k_2_ (L^2^·mol^−2^)
1,2-ethylene glycol	2.15	1.15
1,3-propanediol	1.27	0.11
Glycerin	16.0	41.2
Catechol	7.8 × 10^3^	1.42 × 10^4^
D-mannitol	1.10 × 10^2^	1.37 × 10^5^
D-glucose	1.5 × 10^3^	7.60 × 10^3^
Sorbitol	/	4.4 × 10^5^
D-ribose	/	1.57 × 10^7^

**Table 4:** The dose toxicological effect of boron on animals (Adapted with permission from Ref. [28]. 2018, Springer).

Animals	Dose (mg·kg^−1^)	Toxicological Effect
Mouse	79	Slow growth
Rabbit	44	Fetal deformities
Dog	29	Testicular atrophy
Rat	26	Sperm inhibition
Rat	52	Testicular atrophy
Rat	13	Decreased fetus body size

**Table 5:** The guideline values of boron in drinking water or industrial effluents suggested by different countries or organizations (Adapted with permission from Refs. [2,30,31]. 2021, Elsevier; 2014, CNKI; 2021, Elsevier).

Countries/ Organizations	Drinking Water (mg·L^−1^)	Industrial Effluent (mg·L^−1^)	Countries	Drinking Water (mg·L^−1^)	Industrial Effluent (mg·L^−1^)
WHO	2.4	-	China	0.5	5.0 (Shanghai) 2.0 (Beijing)
EU	1.0	-	Malaysia	0.5	4.0
USA	1.0 (California) 0.9 (Wisconsin) 0.63 (Florida) 0.6 (Minnesota)	-	India	0.5	2.0
Canada	5.0	-	Morocco	0.3	-
New Zealand	1.4	-	Egypt	0.5	-
Australia	4.0	-	Kuwait	0.5	-
South Korea	1.0	-	Saudi Arabia	0.5	-
Japan	1.0	10	Iraq	0.1	-
Singapore	2.4	5.0	Jordan	1.0	-
Israel	0.3	1.5	Brazil	-	5.0

**Table 6:** Boron removal performances of different membrane processes.

Membrane Processes		Conditions	Removal Rate	Refs.
RO	UiO-66 + RO	55 bar, 25 °C, pH = 8, [B]_0_ = 5 ppm	91.2%	[37]
RO	NBS + RO	55 bar, 25 °C, pH = 8, [B]_0_ = 5 ppm	93.1%	[13]
RO	PIB/MPD/TMC + RO	1.55 MPa, 25 °C, [PIB] = 0.30%, [B]_0_ = 5 ppm	93.12%	[39]
RO	EDBSA/TMC + RO	1.2 MPa, 25 °C, [EDBSA] = 1%, [TMC] = 0.15%, [B]_0_ = 5 ppm	90.6%	[40]
FO	FTS H_2_O^TM^ membrane	[FS] ^a^: pH = 10, [B]_0_ = 50 mg·L^−1^, [DS] ^b^ = 1 M MgCl_2_	90%	[43]
FO	PSU membrane	[FS]: pH = 10, [B]_0_ = 50 mg·L^−1^, [DS] = 1 M MgCl_2_	84%	[43]
FO	Aquaporin Inside™ membrane	[FS]: pH = 10, [B]_0_ = 50 mg·L^−1^, [DS] = 1 M MgCl_2_	76%	[43]
FO	-	[FS]: pH = 8, [B]_0_ = 10 mg·L^−1^, [DS]: 0.2 M NaCl, pH = 12.5	94%	[44]
ED	M_50_-QGO1 membranes	30 V, 3 h, pH = 9.14, [B]_0_ = 1000 mg·L^−1^	76.6%	[46]
ED	BPED	12.5 V, 60 min, pH = 9.2, [B]_0_ = 100 mg·L^−1^	90.2%	[48]
DD	AFN	[B]_0_ = 66 mg·L^−1^, pH = 11.6, [Cl^−1^] = 0.5 mg·L^−1^	88.8%	[50]
MD	PVDF membrane	205 kPa,18 h, 59 °C, pH = 7.48, [B]_0_ = 5.37 mg·L^−1^	91.25%	[27]
MD	PVDF membrane	180 kPa, 250 h, 50 °C, pH = 7.7, [B]_0_ = 12.7 mg·L^−1^	99.8%	[27,52]

^a^ [FS]: feed solution; ^b^ [DS]: draw solution.

**Table 8:** Characteristics of different commercial resins and fibers.

Commercial Resin/Fiber	Manufacturer	Adsorption Capacity	Refs.
Diaion CRB01	Mitsubishi Chemical Corporation	≥1.2 eq·L^−1^	[7]
Diaion CRB02	Mitsubishi Chemical Corporation	7.46 mg·g^−1^	[8]
Diaion CRB03	Mitsubishi Chemical Corporation	≥0.7 eq·L^−1^	[65]
Diaion CRB05	Mitsubishi Chemical Corporation	≥0.95 eq·L^−1^	[65]
Dowex 2 × 8	Dow Chemical Company	17.0 mg·g^−1^	[66]
Dowex XUS 43594.00	Dow Chemical Company	3.35 mg·g^−1^	[8]
Dowex^TM^ BSR-1	Dow Chemical Company	0.7 eq·L^−1^	[7]
Amberlite IRA-743	Rohm & Haas Company	7.46 mg·g^−1^	[8]
Amberlite PWA10	Rohm & Haas Company	≥0.7 eq·L^−1^	[7]
Purolite S108	Purolite Company	6.27 mg·g^−1^	[8]
Purolite S110	Purolite Company	0.8 eq·L^−1^	[7]
Chelest fiber GRY-HW	Chelest Company	12.07 mg·g^−1^	[67]

**Table 9:** Characteristics of several modified resins based on NMDG resins.

Modified Resins	Functional Monomer	Saturated Adsorption Capacity	Refs.
Glycidyl methacrylate-NMDG	NMDG	20.00 mg·g^−1^	[75]
PAF-1-NMDG P2-NMDG	NMDG	18.38 mg·g^−1^ 16.86 mg·g^−1^	[72] [72]
CA@KH-550@EPH@ NMDG(CKEN)	NMDG	15.35 mg·g^−1^	[74]
3DOM CLPGMA-NMDG-6	NMDG	24.00 mg·g^−1^	[78]

**Table 10:** Boron removal performance of LDHs adsorbents.

Adsorbent	Conditions	Equilibrium ^a^/Maximum ^b^ Adsorption Capacity	Refs.
CQDs/LDHs	[B]_0_ = 25 mg·L^−1^, 3 h, pH = 8.5, adsorbent dose = 2 g·L^−1^, 25 °C	^a^ 19.5 mg·g^−1^	[79]
Perlite-HDTMA	[B]_0_ = 8000 mg·L^−1^, 4 h, pH = 4, 25 °C	^b^ 833.3 mg·g^−1^	[80]
Perlite-GA	[B]_0_ = 8000 mg·L^−1^, 15 h, pH = 7–9, 25 °C	^b^ 2500 mg·g^−1^	[80]
FHT	[B]_0_ = 25 mg·L^−1^, 1.5 h, adsorbent dose = 4 g·L^−1^, 25 °C	^a^ 3.1 mg·g^−1^	[81]
I-LDH	[B]_0_ = 1000 mg·L^−1^, 24 h, pH = 7, adsorbent dose = 7.5 g·L^−1^, 25 °C	^b^ 21.62 mg·g^−1^	[82]
I-CLDH	[B]_0_ = 1000 mg·L^−1^, 24 h, pH = 7, adsorbent dose = 7.5 g·L^−1^, 25 °C	^b^ 77.83 mg·g^−1^	[82]

^a^: The equilibrium adsorption capacity is the adsorption capacity when the adsorption rate is equal to the desorption rate. ^b^: The maximum adsorption capacity is the ideal adsorption capacity that all adsorption sites are filled with adsorbate.

**Table 11:** Boron removal performances of waste industrial materials adsorbents.

Adsorbent	Conditions	Equilibrium ^a^/Maximum ^b^ Adsorption Capacity	Refs.
Fly ash zeolite	[B]_0_ = 50 mg·L^−1^, 0.5 h, pH = 7, adsorbent dose = 20 g·L^−1^, 25 °C	^a^ 2.3 mg·g^−1^	[84]
Waste tire rubber	[B]_0_ = 17.5 mg·L^−1^, 48 h, pH = 2, adsorbent dose = 1 g·L^−1^, 21 °C	^a^ 16.72 mg·g^−1^	[85]
Waste concrete	[B]_0_ = 10 mg·L^−1^, 24 h, pH = 12, adsorbent dose = 66.7 g·L^−1^	^a^ 0.117 mg·g^−1^	[56]
Non-activated waste sepiolite	[B]_0_ = 600 mg·L^−1^, 24 h, pH = 10, adsorbent dose = 2 g·L^−1^, 20 °C	^b^ 96.15 mg·g^−1^	[86]
Activated waste sepiolite	[B]_0_ = 600 mg·L^−1^, 24 h, pH = 10, adsorbent dose = 2 g·L^−1^, 20 °C	^b^ 178.57 mg·g^−1^	[86]
Steelmaking slag	[B]_0_ = 500 mg·L^−1^, 24 h, adsorbent dose = 2 g·L^−1^, 25 °C	^b^ 145 mg·g^−1^	[87]

^a^: The equilibrium adsorption capacity is the adsorption capacity when the adsorption rate is equal to the desorption rate. ^b^: The maximum adsorption capacity is the ideal adsorption capacity that all adsorption sites are filled with adsorbate.

**Table 12:** Performances of natural materials adsorbents in boron adsorption.

Adsorbent	Conditions	Equilibrium ^a^/Maximum ^b^ Adsorption Capacity	Refs.
Bentonite	[B]_0_ = 120 mg·L^−1^, pH = 9, 24 h, [CaCl_2_] = 0.1 M, adsorbent dose = 50 g·L^−1^	^b^ 0.51 mg·g^−1^	[88]
Bentonite-FeCl_3_	[B]_0_ = 120 mg·L^−1^, pH = 9, 24 h, [CaCl_2_] = 0.1 M, adsorbent dose = 50 g·L^−1^	^b^ 0.83 mg·g^−1^	[88]
Kaolinite	[B]_0_ = 120 mg·L^−1^, pH = 9, 24 h, [CaCl_2_] = 0.1 M, adsorbent dose = 50 g·L^−1^	^b^ 0.60 mg·g^−1^	[88]
Kaolinite-FeCl_3_	[B]_0_ = 120 mg·L^−1^, pH = 9, 24 h, [CaCl_2_] = 0.1 M, adsorbent dose = 50 g·L^−1^	^b^ 0.80 mg·g^−1^	[88]
Waste calcite	[B]_0_ = 120 mg·L^−1^, pH = 9, 24 h, [CaCl_2_] = 0.1 M, adsorbent dose = 50 g·L^−1^	^b^ 1.05 mg·g^−1^	[88]
Waste calcite-FeCl_3_	[B]_0_ = 120 mg·L^−1^, pH = 9, 24 h, [CaCl_2_] = 0.1 M, adsorbent dose = 50 g·L^−1^	^b^ 1.60 mg·g^−1^	[88]
Zeolite	[B]_0_ = 120 mg·L^−1^, pH = 9, 24 h, [CaCl_2_] = 0.1 M, adsorbent dose = 50 g·L^−1^	^b^ 0.53 mg·g^−1^	[88]
Zeolite-FeCl_3_	[B]_0_ = 120 mg·L^−1^, pH = 9, 24 h, [CaCl_2_] = 0.1 M, adsorbent dose = 50 g·L^−1^	^b^ 0.76 mg·g^−1^	[88]
Rice residue	[B]_0_ = 120 mg·L^−1^, pH = 7, 48 h, [CaCl_2_] = 0.1 M, adsorbent dose = 2 g·L^−1^	^b^ 9.26 mg·g^−1^	[88]
Rice residue-FeCl_3_	[B]_0_ = 120 mg·L^−1^, pH = 7, 48 h, [CaCl_2_] = 0.1 M, adsorbent dose = 2 g·L^−1^	^b^ 9.17 mg·g^−1^	[88]
Walnut shell residue	[B]_0_ = 120 mg·L^−1^, pH = 7, 48 h, [CaCl_2_] = 0.1 M, adsorbent dose = 2 g·L^−1^	^b^ 7.04 mg·g^−1^	[88]
Walnut shell residue-FeCl_3_	[B]_0_ = 120 mg·L^−1^, pH = 7, 48 h, [CaCl_2_] = 0.1 M, adsorbent dose = 2 g·L^−1^	^b^ 7.58 mg·g^−1^	[88]
Wheat residue	[B]_0_ = 120 mg·L^−1^, pH = 7, 48 h, [CaCl_2_] = 0.1 M, adsorbent dose = 2 g·L^−1^	^b^ 5.59 mg·g^−1^	[88]
Wheat residue-FeCl_3_	[B]_0_ = 120 mg·L^−1^, pH = 7, 48 h, [CaCl_2_] = 0.1 M, adsorbent dose = 2 g·L^−1^	^b^ 6.06 mg·g^−1^	[88]
Magnesite and bentonite clay composite	[B]_0_ = 20 mg·L^−1^, 30 min, pH = 11, adsorbent dose = 2 g·L^−1^, 26 °C	^b^ 4 mg·g^−1^	[89]
Vermiculite-HDTMA	[B]_0_ = 7205 mg·L^−1^, pH = 11, 15 h, 56.5 °C	^a^ 258.13 mg·g^−1^	[90]
Vermiculite-GA	[B]_0_ = 7181.3 mg·L^−1^, pH = 8.48, 2 h, 40.8 °C	^a^ 152.4 mg·g^−1^	[90]
Perlite-HDTMA	[B]_0_ = 8000 mg·L^−1^, 4 h, pH = 4, 25 °C	^b^ 833.3 mg·g^−1^	[80]
Perlite-GA	[B]_0_ = 8000 mg·L^−1^, 15 h, pH = 7–9, 25 °C	^b^ 2500.0 mg·g^−1^	[80]
CWES	[B]_0_ = 50 mg·L^−1^, pH = 4, 48 h, adsorbent dose = 1 g·L^−1^, 25 °C	^b^ 31.06 mg·g^−1^	[57]
ESM	[B]_0_ = 50 mg·L^−1^, pH = 8, 48 h, adsorbent dose = 1 g·L^−1^, 25 °C	^b^ 33.3 mg·g^−1^	[91]
MESM	[B]_0_ = 50 mg·L^−1^, pH = 4, 48 h, adsorbent dose = 1 g·L^−1^, 25 °C	^b^ 33.3 mg·g^−1^	[91]

^a^: The equilibrium adsorption capacity is the adsorption capacity when the adsorption rate is equal to the desorption rate. ^b^: The maximum adsorption capacity is the ideal adsorption capacity that all adsorption sites are filled with adsorbate.

**Table 13:** Boron removal performances of POPs adsorbents.

Adsorbent	Conditions	Maximum Adsorption Capacity	Refs.
ZIF-8	[B]_0_ = 0.5 M, 12 h, pH = 4.43, adsorbent dose = 5 g·L^−1^, 45 °C	247.44 mg·g^−1^	[92]
ZIF-67	[B]_0_ = 0.5 M, 24 h, pH = 4, adsorbent dose = 3 g·L^−1^, 35 °C	579.80 mg·g^−1^	[93]
UiO-66	[B]_0_ = 0.7 M, 12 h, adsorbent dose = 5 g·L^−1^, 35 °C	140.53 mg·g^−1^	[94]
PAF-1-NMDG	[B]_0_ = 19.4 mM·L^−1^, 1 h, adsorbent dose = 5 g·L^−1^	18.38 mg·g^−1^	[72]
P2-NMDG	[B]_0_ = 19.4 mM·L^−1^, 1 h, adsorbent dose = 5 g·L^−1^	16.86 mg·g^−1^	[72]
3DOM CLPGMA-NMDG-6	[B]_0_ = 500 mg·L^−1^, pH = 8, 24 h, adsorbent dose = 5 g·L^−1^, 25 °C	24.00 mg·g^−1^	[78]
CTS-NMDG	[B]_0_ = 2000 mg·L^−1^, pH = 7, 10 h, adsorbent dose = 10 g·L^−1^, 25 °C	20.36 mg·g^−1^	[95]

**Table 14:** Performances of metal oxide-based adsorbents in boron adsorption.

Adsorbent	Size (μm)	T (°C)	pH	Maximum Adsorption Capacity	Refs.
MgO	-	30	10	216 mg·g^−1^	[96]
Al_2_O_3_	-	-	9	6.4 mg·g^−1^	[98]
CAAl	800	-	9	56.3 mg·g^−1^	[98]
TiO_2_-CTS	450	25	4	4.35 mg·g^−1^	[99]
Cr_2_O_3_-CTS	450	25	4	3.52 mg·g^−1^	[99]
Fe_3_O_4_-CTS	450	25	4	4.42 mg·g^−1^	[99]

**Table 15:** Boron removal performances of new material adsorbents.

Adsorbent	Conditions	Equilibrium ^a^/Maximum ^b^ Adsorption Capacity	Refs.
M-NMDG	[B]_0_ = 32 mg·L^−1^, 30 min, pH = 8.2 adsorbent dose = 1.2 g·L^−1^, 25 °C	^b^ 6.68 mg·g^−1^	[100]
M-TACA	[B]_0_ = 32 mg·L^−1^, 30 min, pH = 8.2 adsorbent dose = 1.2 g·L^−1^, 25 °C	^b^ 13.44 mg·g^−1^	[100]
poly(β-CD-(NH_2_)_7_-TCL)@gluconolactone	[B]_0_ = 300 mg·L^−1^, pH = 9.2 adsorbent dose = 2 g·L^−1^, 25 °C	^a^ 26.3 ± 5.9 mg·g^−1^	[101]
poly(β-CD-(NH_2_)_7_-TFN)@gluconolactone	[B]_0_ = 300 mg·L^−1^, pH = 9.2 adsorbent dose = 2 g·L^−1^, 25 °C	^a^ 44.0 ± 1.8 mg·g^−1^	[101]
β-CD-9PGMA-NMDG	[B]_0_ = 1000 mg·L^−1^, 1 h, pH = 8, adsorbent dose = 5 g·L^−1^, 20 °C	^b^ 31.1 mg·g^−1^	[55]
β-CD-9PGMA-EN-PG	[B]_0_ = 1000 mg·L^−1^, 1 h, pH = 8, adsorbent dose = 5 g·L^−1^, 20 °C	^b^ 20.5 mg·g^−1^	[55]
P(GMA-co-TRIM)-EN-PG	[B]_0_ = 1000 mg·L^−1^, pH = 9, adsorbent dose = 5 g·L^−1^, 30 °C	^a^ 29.2 mg·g^−1^	[102]
P(GMA-co-TRIM)-TETA-PG	[B]_0_ = 1000 mg·L^−1^, pH = 8, adsorbent dose = 5 g·L^−1^, 30 °C	^a^ 23.3 mg·g^−1^	[102]
Zr-CTS	[B]_0_ = 500 mg·L^−1^,48 h, pH = 7, adsorbent dose = 100 g·L^−1^, 25 °C	^a^ 24.5 mg·g^−1^	[58]
HSGUM	[B]_0_ = 25 M, 24 h, 25 °C, adsorbent dose = 5 g·L^−1^	^b^ 44.32 mg·g^−1^	[103]
CKEN	[B]_0_ = 100 M,15 h, pH = 9.5, 25 °C adsorbent dose = 12 g·L^−1^	^b^ 31.8 mg·g^−1^	[74]

^a^: The equilibrium adsorption capacity is the adsorption capacity when the adsorption rate is equal to the desorption rate. ^b^: The maximum adsorption capacity is the ideal adsorption capacity that all adsorption sites are filled with adsorbate.

**Table 16:** Boron removal performances of chemical precipitation and (electric)coagulation.

Processes		Conditions	Removal Rate	Refs.
CP	Lime milk	[B]_0_ = 31.5 g·L^−1^, pH = 10, lime milk dosage = 30 g·L^−1^	71.4%	[107]
COP	H_2_O_2_ + Ba(OH)_2_	[B]_0_ = 1000 mg·L^−1^, 4 h, pH = 10.5	99.7%	[110]
CC	PACSM	[B]_0_ = 5 mg·L^−1^, pH = 10.5, 15 °C, [PACSM] = 0.5 mg·L^−1^	87.5%	[128]
CC	PAFCS	[B]_0_ = 5 mg·L^−1^, pH = 11, 20 °C, [PAFCS] = 3 mg·L^−1^	93.6%	[128]
EC	EC-Al	[B]_0_ = 100 mg·L^−1^, D * = 10 mm, pH = 8, 60 min, CD = 5.5 mA·cm^−2^	70%	[114]
EC	EC-Al	[B]_0_ = 9.3 mM, [NaCl] = 10 mM, pH = 8.0, CD = 5 mA·cm^−2^	74.1%	[121]
EC	EC-Al	[B]_0_ = 500 mg·L^−1^, pH = 8.5, 90 min, CD = 10 mA·cm^−2^	55%	[116]
EC	EC-Ni	[B]_0_ = 10 mg·L^−1^, pH = 8, 2 h, CD = 1.25 mA·cm^−2^	99.2%	[117]
EC	EC-Fe/Ni	[B]_0_ = 10 mg·L^−1^, pH = 8, 60 min, CD = 3.75 mA·cm^−2^	95%	[118]
EC	EC-Al	[B]_0_ = 15 mg·L^−1^, pH = 8, 150 min, CD = 6 mA·cm^−2^	96%	[124]
EC	EC-Al	[B]_0_ = 10.4 mg·L^−1^, pH = 6.3, 89 min, CD = 17.4 mA·cm^−2^	99.7%	[129]
EC	EC-Al	[B]_0_ = 5 mg·L^−1^, 45 min, D = 5 mm, pH = 7.84, CD = 12.5 mA·cm^−2^	88%	[125]
EC	EC-Fe	[B]_0_ = 5 mg·L^−1^, 45 min, D = 5 mm, pH = 7.84, CD = 12.5 mA·cm^−2^	78%	[125]

* D: distance between electrodes.

**Table 17:** Boron removal performances of different extractants.

Extractant	Conditions	Extraction Rate	Refs.
EHD/CTMP	0.25 M EHD/0.25 M CTMP, pH = 9.2	90.0%	[130]
BPO	[E] * = 0.1 M, [B]_0_ = 0.1 M	63.1%	[139]
4,5-Dimethyl-2,4-hexanediol	[B]_0_ = 0.01 M, [E] = 0.5 M, 25 °C, pH = 2	85.9%	[133]
4,6-Dimethyl-2,4-heptanediol	[B]_0_ = 0.01 M, [E] = 0.5 M, 25 °C, pH = 2	71.2%	[133]
4,7-Dimethyl-2,4-octanediol	[B]_0_ = 0.01 M, [E] = 0.5 M, 25 °C, pH = 2	68.8%	[133]
2,2,4-Trimethyl-1,3-pentanediol	[B]_0_ = 0.01 M, [E] = 0.5 M, 25 °C, pH = 2	96.9%	[133]
2,2,5-Trimethyl-1,3-hexanediol	[B]_0_ = 0.01 M, [E] = 0.5 M, 25 °C, pH = 2	96.8%	[133]
2,2,6-Trimethyl-1,3-heptanediol	[B]_0_ = 0.01 M, [E] = 0.5 M, 25 °C, pH = 2	91.4%	[133]
2,3,4-Trimethyl-1,3-pentanediol	[B]_0_ = 0.01 M, [E] = 0.5 M, 25 °C, pH = 2	96.3%	[133]
2,3,5-Trimethyl-1,3-hexanediol	[B]_0_ = 0.01 M, [E] = 0.5 M, 25 °C, pH = 2	96.2%	[133]
2,3,6-Trimethyl-1,3-heptanediol	[B]_0_ = 0.01 M, [E] = 0.5 M, 25 °C, pH = 2	94.8%	[133]
2-butyl-2-ethyl-l,3-propanediol	[B]_0_ = 0.03 M, [E] = 1.1 M	78.0%	[135]
2-ethylhexanol	-	99.5%	[138]
2-butyl-1-n-octanol	[B]_0_ = 14.84 g·L^−1^, [E] = 0.2 M, O/A = 1	99.4%	[136]
2-ethyl-1,3-hexanediol in toluene	[B]_0_ = 0.1748 M, 45 °C, pH = 1,	93.5%	[134]
BEPD in 25% decanol/Kerosene	[B]_0_ = 148 mg·L^−1^, [E] = 0.6 M	85.0%	[132]
TMPD in 25% decanol/Kerosene	[B]_0_ = 148 mg·L^−1^, [E] = 0.6 M	93.0%	[132]
2-ethyl-1-hexanol in kerosene	[B]_0_ = 2 g·L^−1^, [E] = 70%	98.3%	[131]
2-ethylhexanol in kerosene	[B]_0_ = 7.29 g·L^−1^, pH = 1.54, [E] = 50%, O/A = 4,	98.8%	[137]

* [E]: Extractant concentration/volume fraction.

## Data Availability

Not applicable.
